# The effect of gradient nonlinearities on fiber orientation estimates from spherical deconvolution of diffusion magnetic resonance imaging data

**DOI:** 10.1002/hbm.25228

**Published:** 2020-10-09

**Authors:** Fenghua Guo, Alberto de Luca, Greg Parker, Derek K. Jones, Max A. Viergever, Alexander Leemans, Chantal M. W. Tax

**Affiliations:** ^1^ Image Sciences Institute University Medical Center Utrecht, Utrecht University Utrecht The Netherlands; ^2^ Cardiff University Brain Research Imaging Centre (CUBRIC), Cardiff University Cardiff UK

**Keywords:** connectivity matrices, constrained spherical deconvolution, damped Richardson‐Lucy, diffusion MRI, fiber orientation distribution, gradient nonlinearity, spherical deconvolution

## Abstract

Gradient nonlinearities in magnetic resonance imaging (MRI) cause spatially varying mismatches between the imposed and the effective gradients and can cause significant biases in rotationally invariant diffusion MRI measures derived from, for example, diffusion tensor imaging. The estimation of the orientational organization of fibrous tissue, which is nowadays frequently performed with spherical deconvolution techniques ideally using higher diffusion weightings, can likewise be biased by gradient nonlinearities. We explore the sensitivity of two established spherical deconvolution approaches to gradient nonlinearities, namely constrained spherical deconvolution (CSD) and damped Richardson‐Lucy (dRL). Additionally, we propose an extension of dRL to take into account gradient imperfections, without the need of data interpolation. Simulations show that using the effective *b*‐matrix can improve dRL fiber orientation estimation and reduces angular deviations, while CSD can be more robust to gradient nonlinearity depending on the implementation. Angular errors depend on a complex interplay of many factors, including the direction and magnitude of gradient deviations, underlying microstructure, SNR, anisotropy of the effective response function, and diffusion weighting. Notably, angular deviations can also be observed at lower *b*‐values in contrast to the perhaps common assumption that only high *b*‐value data are affected. In in vivo Human Connectome Project data and acquisitions from an ultrastrong gradient (300 mT/m) scanner, angular differences are observed between applying and not applying the effective gradients in dRL estimation. As even small angular differences can lead to error propagation during tractography and as such impact connectivity analyses, incorporating gradient deviations into the estimation of fiber orientations should make such analyses more reliable.

## Graphical abstract

Gradient nonlinearities cause spatially varying mismatches between the imposed and the effective gradients in diffusion magnetic resonance imaging. We explore the sensitivity of spherical deconvolution approaches, including constrained spherical deconvolution and damped Richardson‐Lucy (dRL), to gradient nonlinearity artifacts. Additionally, we propose an extension of the dRL method to take into account gradient imperfections without the need of data interpolation. As even small angular differences can lead to error propagation during fiber tractography, incorporating gradient deviations into the estimation of the fiber orientation distributions should make such analyses more reliable.

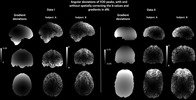

## 1 INTRODUCTION

In magnetic resonance imaging (MRI), encoding of the signal to spatial location and molecular motion is achieved through the application of magnetic field gradients. Due to hardware limitations, however, these gradient fields can be significantly nonuniform throughout the imaging volume, causing the effective gradient amplitude and spatial orientation to deviate from the desired values. Gradient nonlinearities can be characterized from the gradient coil configuration or, when this is not available, by measuring specially designed phantoms (Nagy, Alexander, & Weiskopf, 2009). According to previous works, the gradient deviations become more significant when moving away from the isocenter (Bammer et al., 2003; Glover & Pelc, 1986; Malyarenko, Ross, & Chenevert, 2014; Mohammadi et al., 2012; Nagy, Weiskopf, Alexander, & Deichmann, 2007).

In structural MRI, gradient nonlinearities cause geometric distortions and image intensity inaccuracies, which can significantly affect quantitative group and multisite studies (Jovicich et al., 2006; Tax et al., 2019). Several frameworks have been developed to, retrospectively, correct such geometric distortions (Glover & Pelc, 1986; Jovicich et al., 2006). In diffusion MRI, where magnetic field gradients are used to sensitize the signal to the microscopic motion of spins, gradient nonlinearities additionally result in spatially varying diffusion sensitization (Bammer et al., 2003; Malyarenko et al., 2014; Sotiropoulos et al., 2013). Since the diffusion weighting (often summarized by the *b*‐value) scales quadratically with the gradient amplitude, these effects can be more prominent with ultrastrong gradient systems. Although gradient nonlinearities are often considered to be problematic with strong gradients only, it was shown that even with clinical systems equipped with 40 mT/m gradients, the resulting fields can deviate more than 10% from the expected values at the edges of the volume (Bammer, 2003; Jovicich et al., 2006; Mohammadi et al., 2012; Nagy et al., 2007). In addition, higher gradient strengths are becoming more commonplace in clinical settings and the state‐of‐the‐art Connectom scanner can even achieve gradient strengths up to 300 mT/m (Jones et al., 2018; Setsompop et al., 2013). For this reason, imaging consortiums maintaining public databases (Sudlow et al., 2015; Tax et al., 2019; Van Essen et al., 2012; Van Essen et al., 2013) such as the Human Connectome Project (HCP) have recognized the detrimental effects of gradient nonlinearities on diffusion measures and are now providing additional information to allow for their mitigation.

The effect of gradient nonlinearities in diffusion MRI and their corrections have been investigated in several studies (Bammer et al., 2003; Jovicich et al., 2006; Malyarenko et al., 2014; Mohammadi et al., 2012; Nagy et al., 2007; Setsompop et al., 2013). Water phantom studies (Nagy et al., 2009; Rogers et al., 2018, 2017) reveal gradient field inhomogeneities as one of the main factors contributing to inaccuracies in the estimated apparent diffusion coefficient (ADC). Biases of up to 10% in diffusion measures derived from diffusion tensor imaging (DTI) and diffusion kurtosis imaging (DKI) resulting from gradient field nonlinearities have been reported (Bammer et al., 2003; Mesri, David, Viergever, & Leemans, 2020, 2018). Furthermore, gradient nonlinearities have a direct effect on the accuracy of fiber orientation estimation (Glasser et al., 2013; Setsompop et al., 2013; Sotiropoulos et al., 2013) and therefore also bias any subsequent fiber tractography.

During parameter estimation on diffusion MRI data, the diffusion gradient strength and its orientation are commonly assumed to be constant across all voxels in the dataset. When this condition is not met, a possible correction strategy is to generate a gradient encoding matrix (*b*‐matrix) for each voxel independently. In the ADC‐, DTI‐, or DKI‐equation, among others, the correct spatially varying *b*‐matrix can be naturally integrated, whereas this becomes less trivial for methods which impose certain restrictions on the sampling strategy, such as spherical deconvolution using spherical harmonics (SH) (Tournier, Calamante, & Connelly, 2007) or diffusion spectrum imaging (Wedeen, Hagmann, Tseng, Reese, & Weisskoff, 2005).

Spherical deconvolution formulations are commonly used to resolve the fiber orientation distribution (FOD) function, and often require a specific spherical sampling of q‐space (i.e., “shells” for a specific diffusion weighting). Constrained spherical deconvolution (CSD) (Tournier et al., 2007), for instance, represents the diffusion MRI data in the SH basis to perform the deconvolution operation, which inherently assumes the diffusion weighting to be constant per shell. The same holds for techniques that simultaneously aim at estimating the FOD and the deconvolution kernel, often relying on spherical averaging (or powder averaging) of the signal (Edén, 2003; Kaden, Knösche, & Anwander, 2007; Novikov, Veraart, Jelescu, & Fieremans, 2018; Szczepankiewicz et al., 2016). The compatibility of data affected by gradient nonlinearities with such approaches relies on a radial representation of the signal (Morez, Sijbers, & Jeurissen, 2017; Paquette, Eichner, & Anwander, 2019), e.g., to interpolate data back onto shells. Among the various spherical deconvolution strategies, the damped Richardson‐Lucy (dRL) (Dell'Acqua et al., 2010) does not assume the data to lie on shells and can be used in combination with a voxel specific response function, which can in turn be exploited to investigate the bias caused by gradient field nonlinearities, albeit at the cost of increased computational demands. Even though this still relies on a radial representation of the response function, the data itself are not interpolated, thereby circumventing potential interpolation inaccuracies, fitting issues, and modifying noise properties.

In this work, we study the effect of gradient nonlinearities on estimates of fiber orientation from FODs reconstructed with dRL and CSD. In the case of dRL, we propose a formulation of the deconvolution matrix to account for the effect of gradient nonlinearities and compare the application of dRL with and without correction. Additionally, we suggest a “semicorrection” heuristic for CSD based on the *b*‐matrix, that is, using the average of the true *b*‐values as “shell” for each voxel. We evaluate the methods and the suggested corrections with simulations, and with in vivo human datasets from state‐of‐the‐art acquisitions, including a dataset from the HCP (Van Essen et al., 2012) and a public harmonization dataset from an ultrastrong 300 mT/m gradient system (Tax et al., 2019).

## 2 METHODS

We introduce some background theory on the computation of the effective gradients applied in each voxel in Section 2.1, and then briefly describe the dRL and CSD techniques and the suggested correction schemes. In Section 2.3, we outline the data and experiments, and in Section 2.4, the analysis strategies.

### 2.1 Spatially varying *b*‐matrix

Given the gradient coil tensor ***L***(***r***) for each location ***r*** (where we define Δ***L***(***r***) = ***L***(***r***) − ***I***), the effective gradient ***g***_eff_ and the imposed gradient ***g*** are related by the following expression (Bammer et al., 2003):

(1) $$g_{\mathrm{eff}}\left(r\right)=\left(\begin{array}{ccc} L_{\mathrm{xx}}\left(r\right) & L_{\mathrm{xy}}\left(r\right) & L_{\mathrm{xz}}\left(r\right)\\ L_{\mathrm{yx}}\left(r\right) & L_{\mathrm{yy}}\left(r\right) & L_{\mathrm{yz}}\left(r\right)\\ L_{\mathrm{zx}}\left(r\right) & L_{\mathrm{xy}}\left(r\right) & L_{\mathrm{zz}}\left(r\right) \end{array}\right)g=L\left(r\right)g$$

Accordingly, the effective *b*‐matrix ***B***_eff_(***r***) can be related to the imposed *b*‐matrix ***B*** as

(2) $$B_{\mathrm{eff}}\left(r\right)=L\left(r\right)\mathrm{BL}{\left(r\right)}^{T}$$

Here, the imposed *b*‐value *b* = trace(***B***) and the effective *b*‐value *b*_eff_ = trace(***B***_eff_). In the following, we show how ***B***_eff_(***r***) can be incorporated into two spherical deconvolution frameworks.

### 2.2 Spherical deconvolution strategies

As previously mentioned, most spherical deconvolution methods require a “shell” sampling, where data are acquired in multiple diffusion gradient orientations sampled on a sphere for a given diffusion strength (i.e., *b*‐value). For dRL, this requirement can be relaxed, because the dependency on the diffusion weighting can be explicitly taken into account using a representation of the response function that can vary voxel‐wise. In Section 2.2.1, we briefly describe the dRL algorithm, and present a modified version of dRL to consider the spatially varying *b*‐vectors and *b*‐values. In Section 2.2.2, we similarly present CSD and a modified version that can partially account for the spatially varying *b*‐vectors and *b*‐values.

#### 2.2.1 dRL deconvolution

The deconvolution response function used in dRL is represented by ***H*** that maps the diffusion MRI signals onto the FOD. *H* is an *m* × *n* matrix where every column of length *m* contains the values of the fiber response profile oriented along one of the *n*‐directions. In the original dRL method, the *H*‐matrix is generated once and subsequently used for all voxels. In the rest of the manuscript, this method will be referred to as dRL with a uniform *H*‐matrix (*dRL‐uni*).

The FOD of each voxel can then be estimated from the diffusion MRI signal (***S***) through an iterative process estimating the maximum expectation, where k represents the *k*th iteration:

(3) $$f^{\left(k+1\right)}=f^{\left(k\right)}\left(1+u^{\left(k\right)}\frac{H^{T}S-H^{T}Hf^{\left(k\right)}}{H^{T}Hf^{\left(k\right)}}\;\right)$$

Here, the transpose of ***H*** is written as ***H***^*T*^. ***f*** is a column vector which contains the values of the FOD along *n* directions uniformly distributed on a sphere, and *u* is a *n* × 1 vector that performs a damping operation on ***f***.

In the original dRL formulation, the response of a single fiber population was represented by a diffusion tensor corresponding to eigenvalues [*λ*, *β*, *β*] (the second and third eigenvalues are set to be equal), as shown in Equation (4):

(4) $$H_{\mathrm{ij}}=\exp\left(b_{i}\left(\lambda \;\cos^{2}\left(\theta_{\mathrm{ij}}\right)+\beta \left(1-\cos^{2}\left(\theta_{\mathrm{ij}}\right)\right)\right)\right)$$

In Equation (4), *θ*_*ij*_ is the polar angle between the *i*th unit gradient direction of the signal (denoted as $\hat{g}=g/{\left|g\right|}_{2}$) and *j*th FOD orientation sampled on the unit sphere, and *b*_*i*_ is the *b*‐value corresponding to the *i*th gradient direction.

In the modified version of the dRL method introduced in this work (*dRL‐mod*), we propose to compute a voxel‐wise H‐matrix by taking into account the effective *b*‐values and *b*‐vectors experienced at each voxel location, by using the gradient coil tensor ***L***(***r***) as described in Section 2.1. In this case, the H‐matrix at location ***r*** can be written as

(5) $$H_{\mathrm{ij}}\left(r\right)=\exp\left(b_{i,\mathrm{eff}}\left(r\right)\left(\lambda \;\cos^{2}\left(\theta_{\mathrm{ij}}\left(r\right)\right)+\beta \left(1-\cos^{2}\left(\theta_{\mathrm{ij}}\left(r\right)\right)\right)\right)\right)$$

where *θ*_*ij*_(***r***) and *b*_*i*,eff_(***r***) are calculated from the effective *b*‐matrix (which in turn is computed as in Equation (2)). The results in this study were generated with a dRL implementation in MATLAB based on previous studies (Dell'Acqua et al., 2010) with 50 iterations; for peak detection an SH‐fit truncated at order 8 was used (Jeurissen, Leemans, Jones, Tournier, & Sijbers, 2011).

#### 2.2.2 Constrained spherical deconvolution

In CSD, diffusion MRI signals are typically collected for a set of *b*‐vectors (with direction ***g*** and magnitude *b*) on a given shell, and SH coefficients are fitted to the signals for the deconvolution process. To take the effect of nonshelled *b*‐vectors into account, we implemented a modified version of CSD that uses the average effective *b*‐value of each shell. The modified response function then considers the modified ${\hat{g}}_{\mathrm{eff}}$ and averaged *b*‐value ${\bar{b}}_{\mathrm{eff}}$ per shell:

(6) $${\hat{g}}_{i,\mathrm{eff}}=g_{i,\mathrm{eff}}/{\left|g_{i,\mathrm{eff}}\right|}_{2}$$

(7) $${\bar{b}}_{\mathrm{eff}}=\frac{1}{m}\sum_{i=1}^{m}b_{i,\mathrm{eff}}\;$$

CSD with a uniform response function across all voxels will be referred to as *CSD‐uni*, while CSD with a voxel‐wise modified response function using an averaged *b*‐value of all gradient directions will be referred to as *CSD‐mod*. The results in this study were generated by default with CSD implementation in ExploreDTI with SH truncated at order 8, peak threshold of 10% of the average peak amplitude in the iterations and a regularization factor of 1, as suggested in the previous literature (Tournier et al., 2007). For the purpose of comparison, another implementation of CSD in MRtrix (Tournier, Calamante, & Connelly, 2012) with SH truncated at 8 and with both the default settings and modified peak threshold and regularization factors were used when specifically mentioned.

### 2.3 Data

#### 2.3.1 Monte Carlo simulations

In all simulations, the diffusion signals for a single fiber population were represented by a tensor with the following properties (Dell'Acqua et al., 2010): the axial diffusivity was set to 1.7 × 10^−3^ mm^2^/s and the radial diffusivity was set to 0.2 × 10^−3^ mm^2^/s. Signals were generated for 60 directions distributed over half the unit sphere unless indicated otherwise. The simulated signals were fitted with both dRL and CSD.

##### Simulation I: Influence of fiber orientation

A single fiber configuration was generated with the tensor model with the fiber orientation along the x‐, y‐, and z axes, respectively. We simulated a diffusion weighting of *b* = 3,000 s/mm^2^ and 60 gradient orientations. A relative gradient deviation of Δ***L***(***r***) = diag([−0.13, –0.14, –0.05]) was imposed along the primary axis; that is, a gradient deviation that is relatively larger in the x and y axes compared to the z axis (inspired by gradient deviations observed for a Connectom scanner anteriorly in the brain). Then, 10^4^ Rician noise realizations were generated to simulate a final noise level equal to SNR = 30 with respect to the nondiffusion weighted signal.

##### Simulation II: Influence of *b*‐value

The same settings used in Simulation I were used while changing the applied diffusion weighting to *b* = 2,000 s/mm^2^ and *b* = 1,000 s/mm^2^, respectively. Then, 10^4^ Rician noise realizations were simulated with SNR = 30. Additionally, a gradient deviation of opposite sign Δ***L***(***r***) = diag([0.13, 0.14, 0.05]) was imposed along the primary axis with the fiber orientation generated along the x‐axis to further explore the effects of positive gradient deviations.

##### Simulation III: Influence of gradient deviation

The above‐mentioned fiber configuration was generated with the main fiber orientation along the y‐axis, a diffusion weighting of *b* = 3,000 s/mm^2^ and 90 gradient orientations. In this case, we simulated a relative gradient deviation within the range of [−0.2 0] along each axis with step 0.1. Then, 10^4^ Rician noise realizations were simulated with SNR = 30.

##### Simulation IV: Influence of SNR

The fiber configuration explained in Simulation I was simulated with a relative gradient deviation of Δ***L***(***r***) = diag([−0.13, –0.14, –0.05]). Then, 10^4^ Rician noise realizations were generated for each SNR level [10, 20, 30, 40, 50].

##### Simulation V: Crossing fibers

Crossing fibers with a separation angle of [90° 75° 60° 45°] and a signal fraction of [0.5 0.5] of the two fiber populations were simulated. Other parameters were *b* = 3,000 s/mm^2^, 60 gradient orientations, and a gradient deviation of Δ***L***(***r***) = diag([−0.15, –0.15, –0.15]) and Δ***L***(***r***) = diag([0.15, 0.15, 0.15]). Then, 10^4^ Rician noise realizations were generated to achieve an SNR of 30.

#### 2.3.2 Synthetic brain

We created a synthetic brain based on the tensors estimated on a subject from the HCP dataset (van Essen et al., 2012). The ground truth of the fiber orientation in each voxel was assumed to coincide with the first eigenvector of the tensors estimated from the *b* = 1,000 s/mm^2^ shell. The imposed and effective gradients were used to generate diffusion MRI signals. Rician noise was added to create a noise level of SNR 30.

#### 2.3.3 In vivo human brain data

##### Dataset I. HCP diffusion MRI data

The original and the modified dRL were applied to two HCP datasets which included 18 *b* = 0 s/mm^2^ and 90 gradient directions at *b* = 3,000 s/mm^2^. For all the HCP in vivo datasets, preprocessing included motion correction and correction for geometric image deformations due to eddy currents, susceptibility differences, and gradient nonlinearities (Glasser et al., 2013). To correct the *b*‐matrix, the gradient coil tensor was provided for each voxel with the data. A simple gradient nonlinearity correction script is provided with the HCP release (Sotiropoulos et al., 2013).

##### Dataset II. 300 mT/m Connectom diffusion MRI data

The dataset was acquired with a *b*‐value of 3,000 s/mm^2^ applied along 60 gradient directions in addition to 18 nonweighted volume. The imaging resolution was 1.2 × 1.2 × 1.2 mm^3^ (Tax et al., 2019). The preprocessing included correction for subject motion, eddy‐current distortions, EPI distortions (Andersson, Skare, & Ashburner, 2003), and gradient nonlinearity distortions (Glasser et al., 2013). The *b*‐matrices were corrected using spatiotemporal gradient nonlinearity information as described in previous work (Rudrapatna, Parker, Roberts, & Jones, 2018), which also takes intervolume motion into account.

### 2.4 Data analysis

In simulations, FOD peaks were extracted using a Newton descent algorithm (Jeurissen et al., 2011), as implemented in ExploreDTI (Leemans, Jeurissen, Sijbers, & Jones, 2009) for dRL and ExploreDTI and MRtrix (Tournier et al., 2012) for CSD. In single fiber simulations, the primary peak orientation was compared to the ground truth, that is, the first eigenvector of the simulated tensor. In crossing fiber simulations, the angular deviations were calculated between the ground truth orientations and the peaks most aligned to them. The FOD peaks were normalized to the mean amplitude of the primary peak estimated from single fiber populations without gradient deviations for each SD method.

For in vivo data, the angular deviations between the primary FOD peaks from dRL‐uni and the dRL‐mod were calculated. A tensor with eigenvalues [1.7 0.2 0.2] × 10^−3^ mm^2^/s was used in dRL FOD estimation, as suggested by previous work (Dell'Acqua et al., 2010). We used an SH order of 8 in the CSD estimation.

### 2.5 Network analysis

The angular deviations can accumulate along tracts and potentially cause streamlines to end in a different area of grey matter. To investigate this, whole brain deterministic tractography was performed with FOD amplitude threshold of 0.1 and a step size of 1 mm, angle threshold of 30° and a fiber length range of [50 500] mm. Grey matter parcellation was performed using the automated anatomical labeling atlas (Tzourio‐Mazoyer et al., 2002). Structural connectivity matrices (CMs) were calculated in ExploreDTI (Leemans et al., 2009). The difference (ΔCM) of the streamline count and streamline length between using dRL‐uni FOD and dRL‐mod FOD was computed. We visualized the graph with the size of the edges representing the ΔCM of the streamline count and streamline length, and the size of the nodes representing the L2‐norm of the ΔCM to all other nodes.

## 3 RESULTS

**FIGURE 1:**
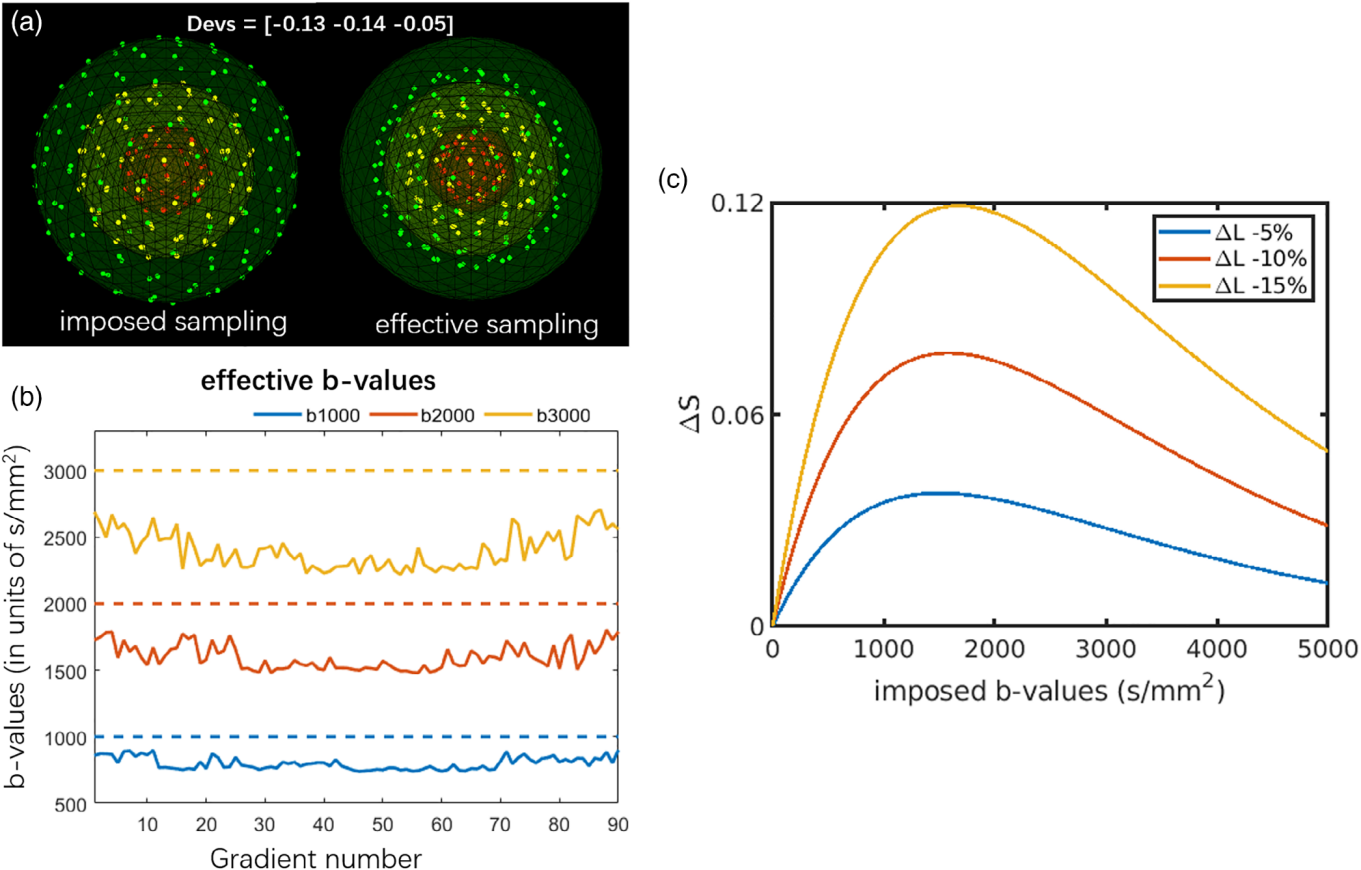
An example of the (a) imposed sampling and the effective sampling with Δ***L***(***r***) = diag ([−0.13, −0.14, −0.05]) with three shells. (b) The effective *b*‐values in each gradient direction (solid line) versus the imposed *b*‐values (dashed line), due to the gradient deviations, the effective sampling does not adhere to “shells.” (c) Absolute signal changes as a function of the imposed *b*‐value in the presence of gradient nonlinearities, that is, *ΔS* = exp(−*bL*^2^*D*) − exp(−*bD*), where a mono‐exponential signal decay was simulated with *D* = 0.7 × 10^−3^ mm^2^/s and *L* = 1 + Δ*L*. The signal deviations depend both on the gradient deviations and the *b*‐values, and the maximum deviation in this scenario occurs at *b* =  − log(1/*L*^2^)/(*D* * (*L*^2^ − 1))

An illustration of the effective *b*‐values and *b*‐vectors experienced in the presence of a gradient deviation of Δ***L***(***r***) = diag([−0.13, −0.14, –0.05]), which was sampled from an in vivo human brain dataset acquired with strong gradients, is shown in Figure 1a,b. Figure 1c shows the absolute signal change in proportion to *S*_0_ for a mono‐exponential signal decay with diffusivity *D* = 0.7 × 10^−3^ mm^2^/s, as a function of the imposed *b*‐value. Larger gradient deviations result in higher signal changes. For a fixed gradient deviation, the largest signal changes are within the range of *b* = 1,500 s/mm^2^ to *b* = 2,000 s/mm^2^ when the gradient deviations Δ***L*** are between −5 and −15%.

### 3.1 Simulations

#### 3.1.1 Simulation I: Influence of fiber orientation

**FIGURE 2:**
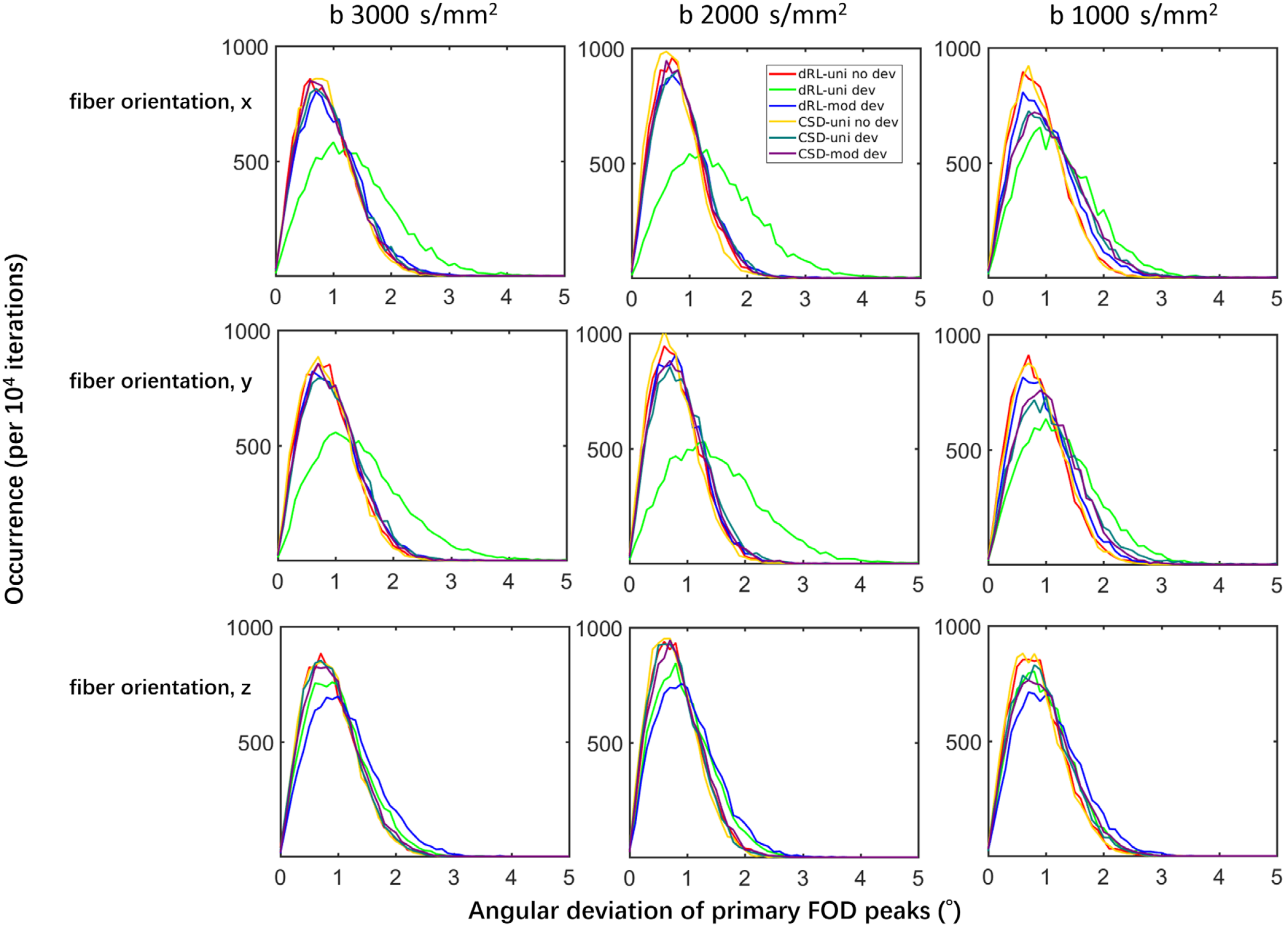
The distribution of the angular deviations of fiber orientation distribution (FOD) peaks from damped Richardson‐Lucy (dRL)‐uni, dRL‐mod, constrained spherical deconvolution (CSD)‐uni, and CSD‐mod estimation of Simulations I and II, with a fixed gradient deviation of Δ***L*** = diag([−0.13 –0.14 −0.05]), at *b* = 3,000 s/mm^2^, *b* = 2,000 s/mm^2^, and *b* = 1,000 s/mm^2^. dRL‐uni no dev: dRL‐uni estimation without the gradient deviation Δ***L***; dRL‐uni dev: dRL‐uni estimation with the gradient deviation Δ***L***; dRL‐mod dev: dRL‐mod estimation with the gradient deviation Δ***L***; CSD‐uni no dev: CSD‐uni estimation without the gradient deviation Δ***L***; CSD‐uni dev: CSD‐uni estimation with the gradient deviation Δ***L***; CSD‐mod dev: CSD‐mod estimation with the gradient deviation Δ***L***

Figure 2 shows the distribution of the estimated FOD peak angular deviations from the ground truth, of dRL‐uni, dRL‐mod, CSD‐uni, and CSD‐mod, with and without gradient nonlinearity effects. Simulations of fibers along x‐, y‐ and z‐ directions are plotted in panels from top to bottom; simulations with *b*‐values of 3,000; 2,000; and 1,000 s/mm^2^, from left to right. Here, we first focus on a comparison between the different fiber orientations, that is, between the rows of Figure 2.

Due to the relatively large gradient deviations in x‐ and y‐ direction and relatively small gradient deviation in z‐direction, larger differences of FOD peak orientation in the x‐ and y‐fibers (top two rows) than in the z‐fibers (bottom row) can be seen. When the fiber is along z‐ direction, the distributions of the angular difference from CSD and dRL estimation are similar, regardless of gradient nonlinearity effects. For the simulations of x‐ and y‐direction fibers, gradient distortions generally increase the median of the angular deviation distribution of FOD peaks over 1° in dRL estimation.

Employing the dRL‐mod framework did improve the angular accuracy of FOD estimation in x‐ and y‐directed fibers where the gradient deviations are over 10%. The distribution of angular deviations of dRL‐mod FOD peaks matches well with the distribution of FODs estimated from signals without any gradient deviations, with medians of the distributions moving from [1.26° 1.31° 1.03°] of [x, y, z] orientation fibers with dRL‐uni estimation to [0.9° 0.87° 0.92°] with dRL‐mod estimation at *b* = 3,000 s/mm^2^ and *b* = 2,000 s/mm^2^. The medians of the angular deviations in FOD estimation are shown in supplementary material Table S1.

CSD slightly outperforms dRL in accuracy of FOD peak orientation estimation, while the angular deviations of CSD FOD peak orientations did not change much with or without the gradient deviation. Modifying the response function did not significantly improve the CSD estimation, with similar angular deviations from the ground truth within approximately 2° and a median of 0.8°. However, the angular deviations with the MRtrix CSD implementation in supplementary Figure S1 top row are showing smaller angular deviations using CSD‐mod compared to CSD‐uni in the presence of negative gradient deviations. At both *b* = 3,000 s/mm^2^ and *b* = 1,000 s/mm^2^, MRtrix CSD‐mod performs better than MRtrix CSD‐uni with negative gradient deviations, while the differences between CSD‐uni and CSD‐mod are not obvious with positive gradient deviations.

#### 3.1.2 Simulation II: Influence of *b*‐value

The columns of Figure 2 show the angular deviations for different *b*‐values. As shown in Figure 1, the averaged effective *b*‐values are around 80% of the imposed *b*‐values. At lower *b*‐values of *b* = 1,000 s/mm^2^ (the right column), the gradient nonlinearity has a more visible effect on the angular deviations of CSD than at higher *b*‐values, whereas the effect remains relatively similar across *b*‐values for dRL. dRL‐mod significantly improves the FOD estimation across *b*‐values when the fiber direction coincides with directions of largest gradient deviations, whereas the improvement of CSD‐mod is less obvious, with the implementation in ExploreDTI. The medians of the angular deviations in the presence of positive gradient deviations in FOD estimation are shown in supplementary material Table S1.

#### 3.1.3 Simulation III: Influence of gradient deviation

**FIGURE 3:**
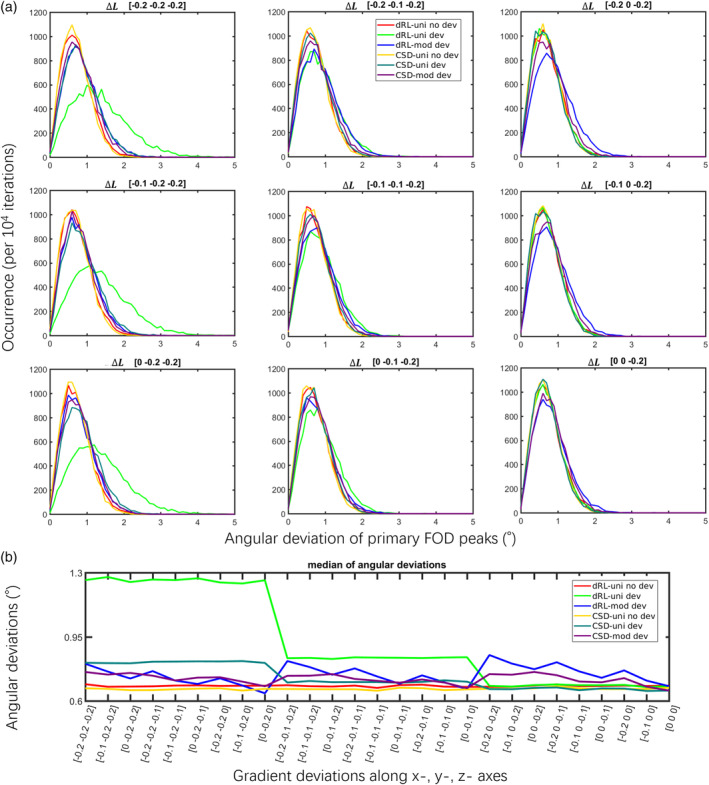
The angular deviations of fiber orientation distribution (FOD) peaks with gradient deviations of Δ***L*** = diag([−0.2 –0.1 0]) in x‐, y‐, and z‐ axes, at SNR 30 of a fixed fiber orientation along the y‐axis (Simulation III), *b* = 3,000 s/mm^2^. Further results of Simulation III with different Δ*L* can be found in supplementary Figure S3. Damped Richardson‐Lucy (dRL)‐uni no dev: dRL‐uni estimation without the gradient deviation Δ***L***; dRL‐uni dev: dRL‐uni estimation with the gradient deviation Δ***L***; dRL‐mod dev: dRL‐mod estimation with the gradient deviation Δ***L***; constrained spherical deconvolution (CSD)‐uni no dev: CSD‐uni estimation without the gradient deviation Δ***L***; CSD‐uni dev: CSD‐uni estimation with the gradient deviation Δ***L***; CSD‐mod dev: CSD‐mod estimation with the gradient deviation Δ***L***

Figure 3a shows the distribution of the estimated FOD peak angular deviations from the ground truth for dRL‐uni, dRL‐mod, CSD‐uni, and CSD‐mod, with and without gradient nonlinearity effects. When varying the gradient deviations while keeping the simulated fiber in y‐direction, dRL‐uni (light green) clearly shows higher angular deviations when the gradient nonlinearities have a large component in the direction of the simulated fiber orientations (the left column). In the case of a gradient deviation of −20% along the fiber orientation (the left bottom), the median of the dRL FOD angular deviations increases from 0.8 to 1.5°, while the range of the distribution increases from 0–3° to 0–5° (light green). In the case of CSD‐uni and CSD‐mod estimation, the distributions of angular deviations are almost identical, with or without gradient deviations. In the extreme circumstances, with a gradient deviation of −20% along the fiber orientation (the left column), CSD‐mod (purple) slightly outperforms CSD‐uni (dark green) in terms of angular deviation. See supplementary Figure S3 for other configurations of Simulation III.

Figure 3b shows the median of the estimated FOD peak angular deviations from the ground truth for dRL‐uni, dRL‐mod, CSD‐uni, and CSD‐mod, with and without gradient nonlinearity effects. When the deviation is along the simulated fiber orientation, that is, the y‐axis in this case, gradient nonlinearities affect the FOD estimation the most, with the median of angular deviations reaching up to 1.3° when the y‐gradient is reduced by 20% for the dRL‐uni estimation (light green).

#### 3.1.4 Simulation IV: Influence of SNR

Similar to the results at SNR 30, dRL‐uni is affected the most compared to other algorithms by gradient nonlinearities for other SNR levels using the regularization settings in this study, with on average 1° larger angular deviations. The distributions of angular deviations are shown in supplementary material Figure S4.

#### 3.1.5 Simulations V: Crossing fibers

**FIGURE 4:**
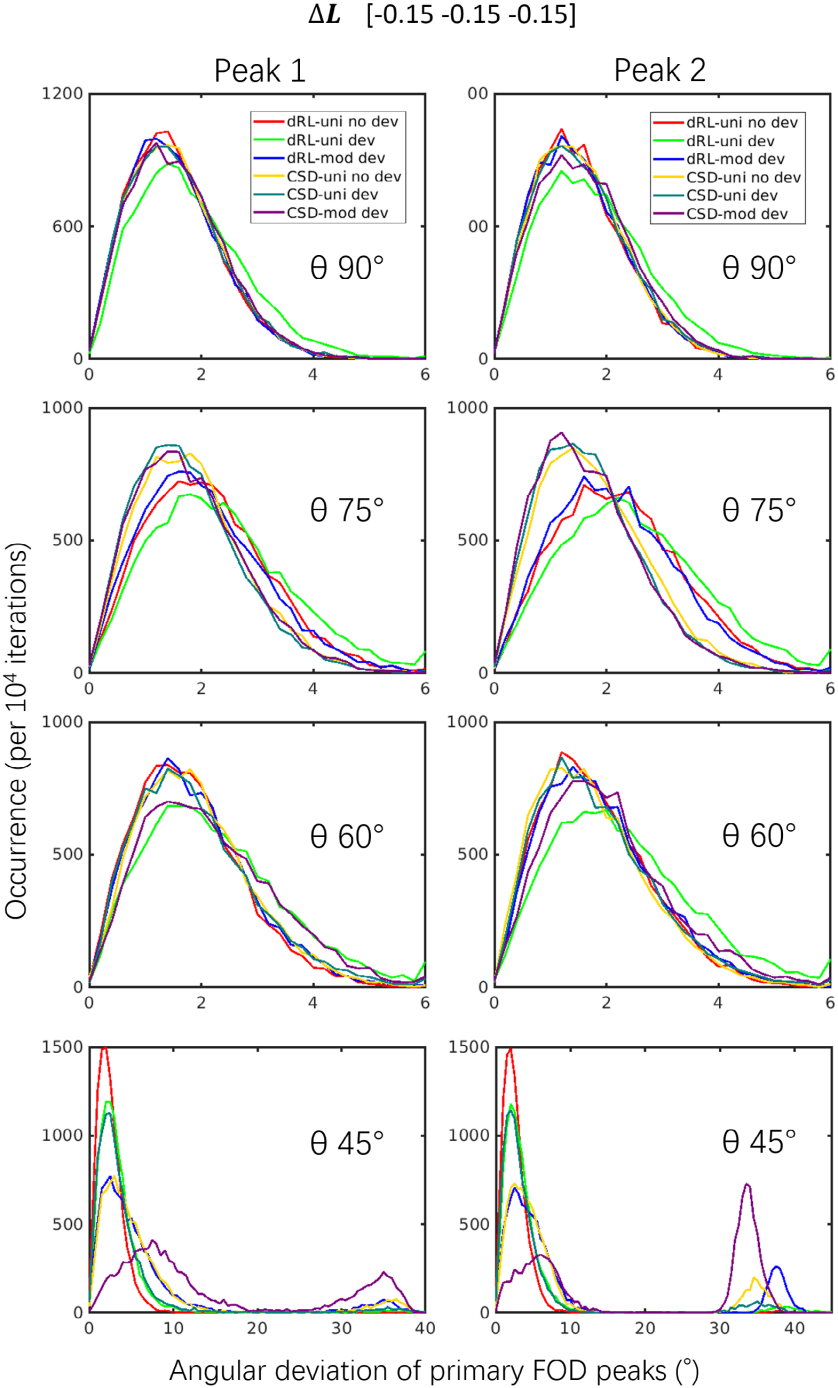
The angular deviations of crossing fibers with Δ***L*** = diag([−0.15 –0.15 –0.15]) (Simulation V), *b* = 3,000 s/mm^2^. *θ* stands for the simulated separation angles of the crossing fibers. Results with a different sign of the diagonal elements in Δ*L* can be found in supplementary Figure S5. Damped Richardson‐Lucy (dRL)‐uni no dev: dRL‐uni estimation without the gradient deviation Δ***L***; dRL‐uni dev: dRL‐uni estimation with the gradient deviation Δ***L***; dRL‐mod dev: dRL‐mod estimation with the gradient deviation Δ***L***; constrained spherical deconvolution (CSD)‐uni no dev: CSD‐uni estimation without the gradient deviation Δ***L***; CSD‐uni dev: CSD‐uni estimation with the gradient deviation Δ***L***; CSD‐mod dev: CSD‐mod estimation with the gradient deviation Δ***L***

Figure 4 shows the angular deviations of dRL‐uni, dRL‐mod, CSD‐uni, and CSD‐mod in crossing fiber simulations. In the presence of large negative gradient deviations (Δ*L* = −15% in along all axes), the angular deviations are relatively stable. At 60, 75, and 90°, dRL‐uni shows the largest angular deviations (green lines). At 75° with the default implementations, CSD performs better than dRL. At 45°, dRL performs better than CSD, that is, resulting in smaller angular deviations. The second “peak” in the histograms of 45° shows that CSD in some cases fails to extract two separate peaks, and CSD‐mod performs worse. On the contrary, with positive gradient deviations as shown in supplementary Figure S5, CSD‐mod performs better than CSD‐uni, which is likely due to the sharper response function used, subsequently improving the angular resolution. See section 4.2 for further discussion.

Figures S6 and S7 show the normalized peak magnitudes. For single fiber populations, dRL‐mod causes larger peaks at negative gradient deviations (Δ***L*** −), and smaller peaks at positive gradient deviations (Δ***L*** +). CSD‐mod results in peak magnitudes that are closer to CSD‐uni without gradient deviations. For crossing fibers, dRL‐mod and CSD‐mod changed the peak magnitudes in the same pattern as for single fibers. CSD shows unstable results at 45° with CSD‐mod at negative gradient deviations (Figure S7, left bottom) and with CSD‐uni at positive gradient deviations (Figure S7, right bottom).

### 3.2 Synthetic brain

**FIGURE 5:**
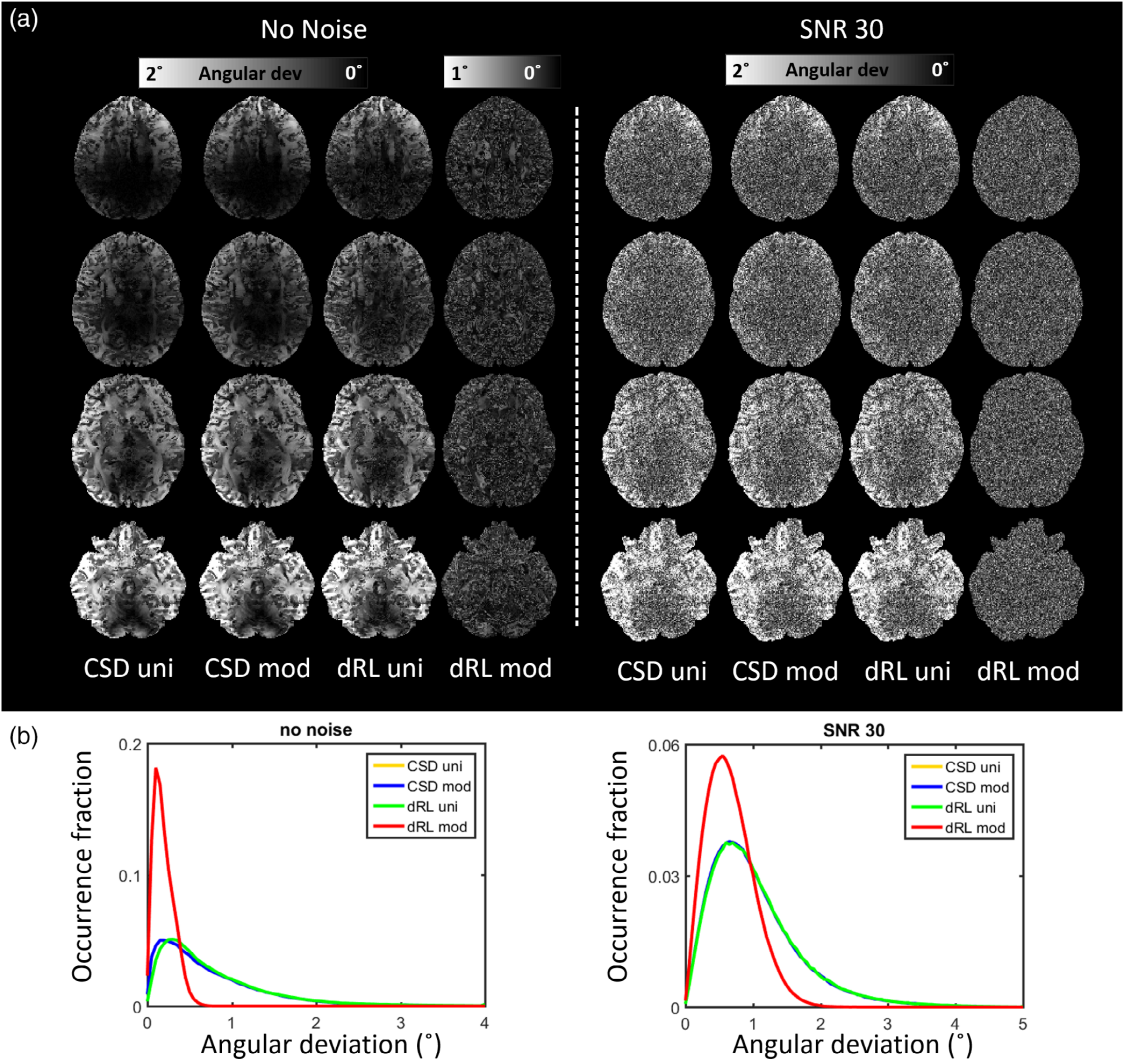
(a) The angular deviation map of the primary fiber orientation distribution (FOD) peak through damped Richardson‐Lucy (dRL)‐uni, dRL‐mod, constrained spherical deconvolution (CSD)‐uni, and CSD‐mod estimation of the synthetic brain from an axial view. (b) The distribution of angular deviations between the estimated primary FOD peak and the simulated fiber orientations. Notice that the yellow lines and green lines are overlapping. CSD (blue and yellow lines) and dRL‐uni (green lines) are close in performance. The angular deviations can reach over 2° toward the edges of the synthetic brain. Using the dRL‐mod FOD estimation (red lines) reduces the angular deviations to within 1° with a medium value of around 0.2°

Figure 5 shows the CSD FOD estimation and the dRL FOD estimation of the synthetic brain both without and with Rician noise (SNR = 30). The angular deviations between the estimated primary FOD peak and the simulated fiber orientations in an axial view are shown in Figure 5a. The fraction of angular deviation occurrences among all the synthetic brain voxels are shown in Figure 5b. Without Rician noise but with gradient field nonlinearity, the dRL‐mod method reduces the angular deviation to less than 1°, with a distribution (red line) much closer to zero than other FOD estimations (i.e., CSD‐uni, CSD‐mod, and dRL‐uni, which are almost overlapping). Ignoring the gradient nonlinearity effects and using uniform *b*‐values and *b*‐vectors, both the CSD estimation (blue line) and dRL estimation (red line) cause an FOD peak angular deviation of up to 3° compared to the ground truth, with a peak centered at 0.2–0.3° (the yellow line overlaps with the green line). At the isocenter of the synthetic brain, the estimated primary fiber orientation matches the ground truth direction more closely, while toward the edges of the gradient field, the angular deviations can reach values over 3°.

**FIGURE 6:**
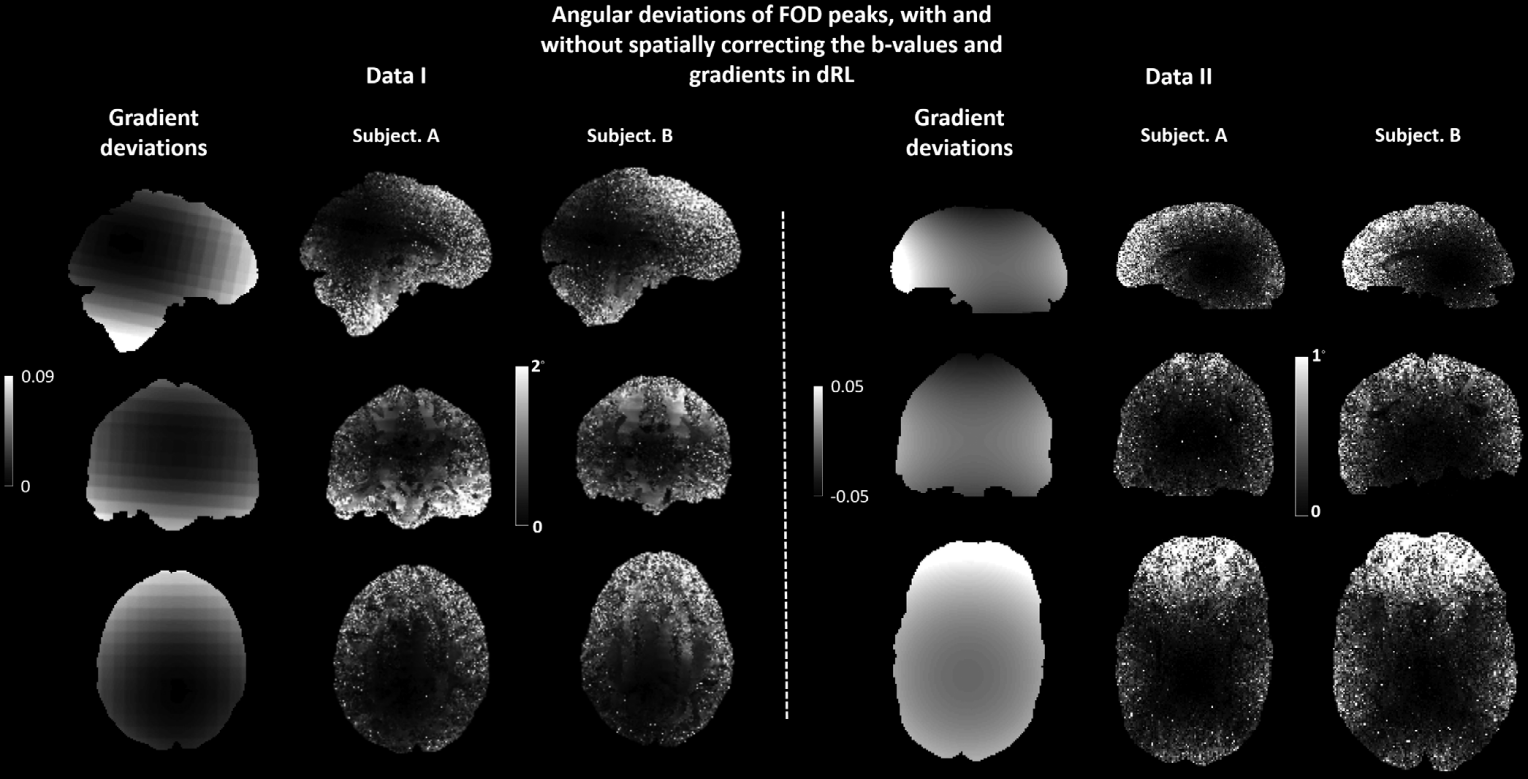
The angular deviations of the primary fiber orientation distribution (FOD) peaks between damped Richardson‐Lucy (dRL)‐uni and dRL‐mod FOD peaks of Data I (Human Connectome Project [HCP] datasets) and Data II (300 mT/m Connectom datasets). The Frobenius norm of the gradient nonlinearity maps within the mask of Subject A in both datasets are plotted next to the angular deviations for visual comparison. For Data I, the high intensity area in the map indicates an angular deviation of above 2°, which mainly occurs in the frontal lobe and the inferior part of the brain. For Data II, a high intensity (above 1°) is observed on the edges of the brain, which is occurring consistently on the frontal lobes. The angular deviations of the subjects follow a spatial pattern that is in line with the gradient field deviations

At SNR 30, the distribution of angular deviations of the modified dRL estimation is likewise moving closer to zero with a narrower peak (red line) compared to the angular deviations of other estimation methods. From an axial view, the angular deviations of the dRL‐mod estimation are much more spatially homogenous, as the effects of gradient nonlinearity are mitigated. From a visual examination, the FOD peak angular deviations of the synthetic brain are globally in correspondence with the Frobenius norm of the coil tensor ***L***(***r***), shown in the first column of Figure 6. Local anatomical structures are visible, indicating a dependence of the angular deviations on the coil tensor and its orientation with respect to the fiber direction. A strong correlation between the magnitude of the gradient deviation and the angular deviation can be seen in frontal, lateral, and inferior regions of the brain, whereas noise dominates gradient nonlinearity effects more medially in the brain.

### 3.3 In vivo human brain data

Figure 6 left shows the angular deviations of the primary FOD peaks between the dRL‐uni and the dRL‐mod estimation of two HCP datasets (Data I). Similar spatial patterns in the angular deviation map can be seen for the two HCP subjects. At the center of the gradient field, only small differences exist between the primary FOD peak orientations of dRL‐uni and dRL‐mod. An angular difference of over 2° can be seen toward the periphery of the brain. Anatomical structures can be identified in some locations, indicating that the angular deviations have a dependency on the underlying fiber orientation.

Figure 6 right shows the angular deviations of the two Connectom MRI datasets (Data II) between the primary FOD peak with the dRL‐uni and dRL‐mod estimation. A similar spatial pattern of angular deviations throughout the brain can be seen in the map of the HCP subjects and Connectome MRI subjects; there is a clear increase in angular deviation from the isocenter toward the periphery of the brain.

**FIGURE 7:**
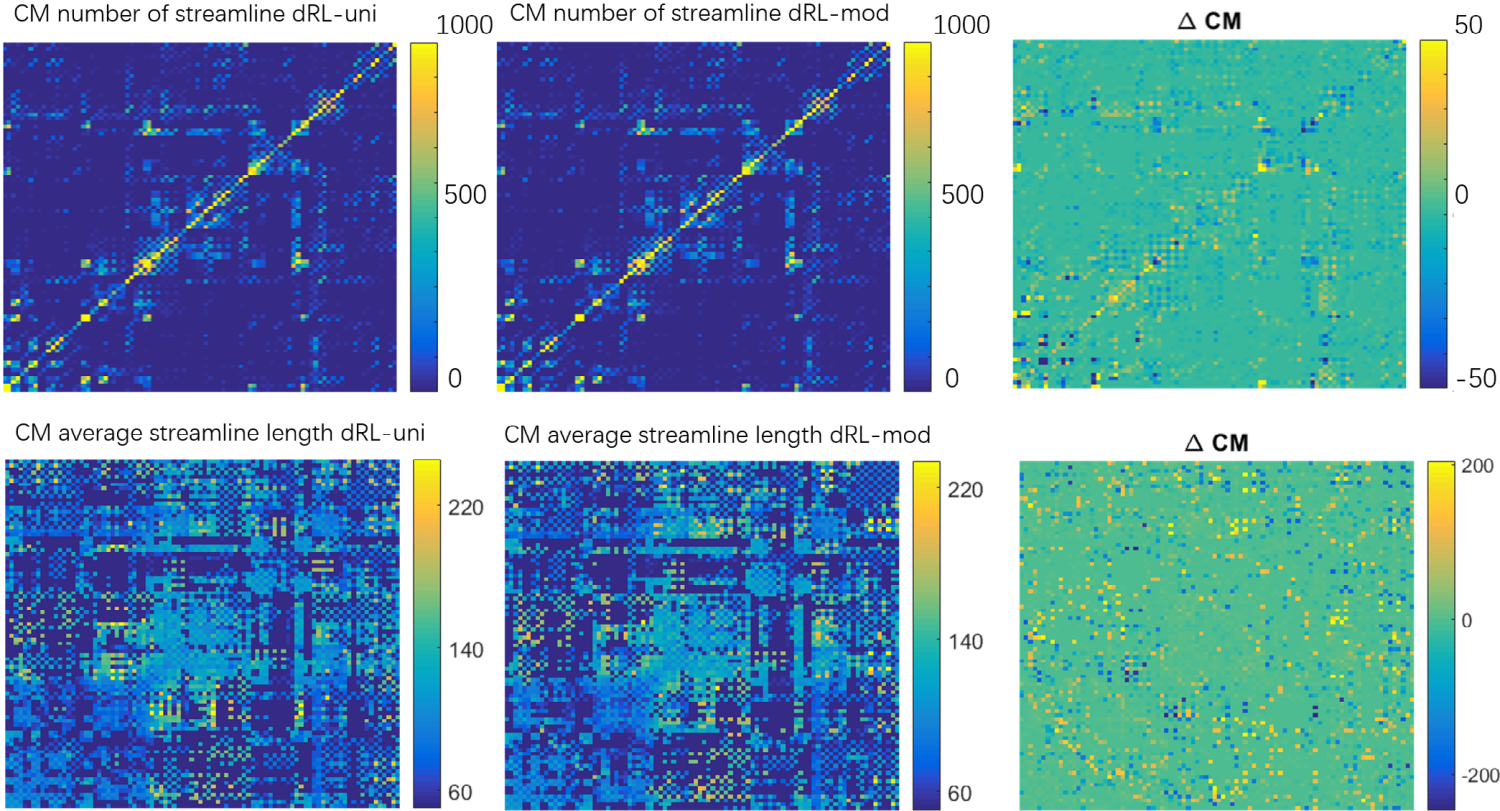
The connectivity matrix (CM) derived from the fiber tractography reconstructed from damped Richardson‐Lucy (dRL)‐uni fiber orientation distributions (FODs) and dRL‐mod FODs, and the differences between dRL‐uni CM and dRL‐mod CM shown in the right column marked as ΔCM. Bright yellow and dark blue areas indicate large differences in the CMs

Figure 7 shows CMs of 90 regions derived from fiber tractography on FODs computed with the dRL‐uni and dRL‐mod estimation. Differences can be observed in the connectivity maps representing the streamline counts (upper row) and average streamline length (bottom row), as shown in the right column marked as ΔCM. A high ΔCM of up to 200 in streamline count indicates that streamlines have potentially deviated into a different cortical region because of the angular deviations caused by gradient nonlinearities in the periphery of the brain. High ΔCM in tract length are also shown in some brain regions, which may be related to differences in termination of tractography. The number of streamlines between the precentral gyrus in the left hemisphere and supplementary motor area in the right hemisphere changes significantly when using dRL‐uni or dRL‐mod FOD estimation. The number of streamlines that starts and ends in the middle frontal gyrus also changes as a result of modifying the voxel‐wise gradients, as well as connections between the supplementary motor area and the superior frontal gyrus. Considering the average tract length, large differences can be found in the CM between regions such as the medial orbital part of the superior frontal gyrus and hippocampus, and between putamen, caudate nucleus and superior occipital gyrus.

**FIGURE 8:**
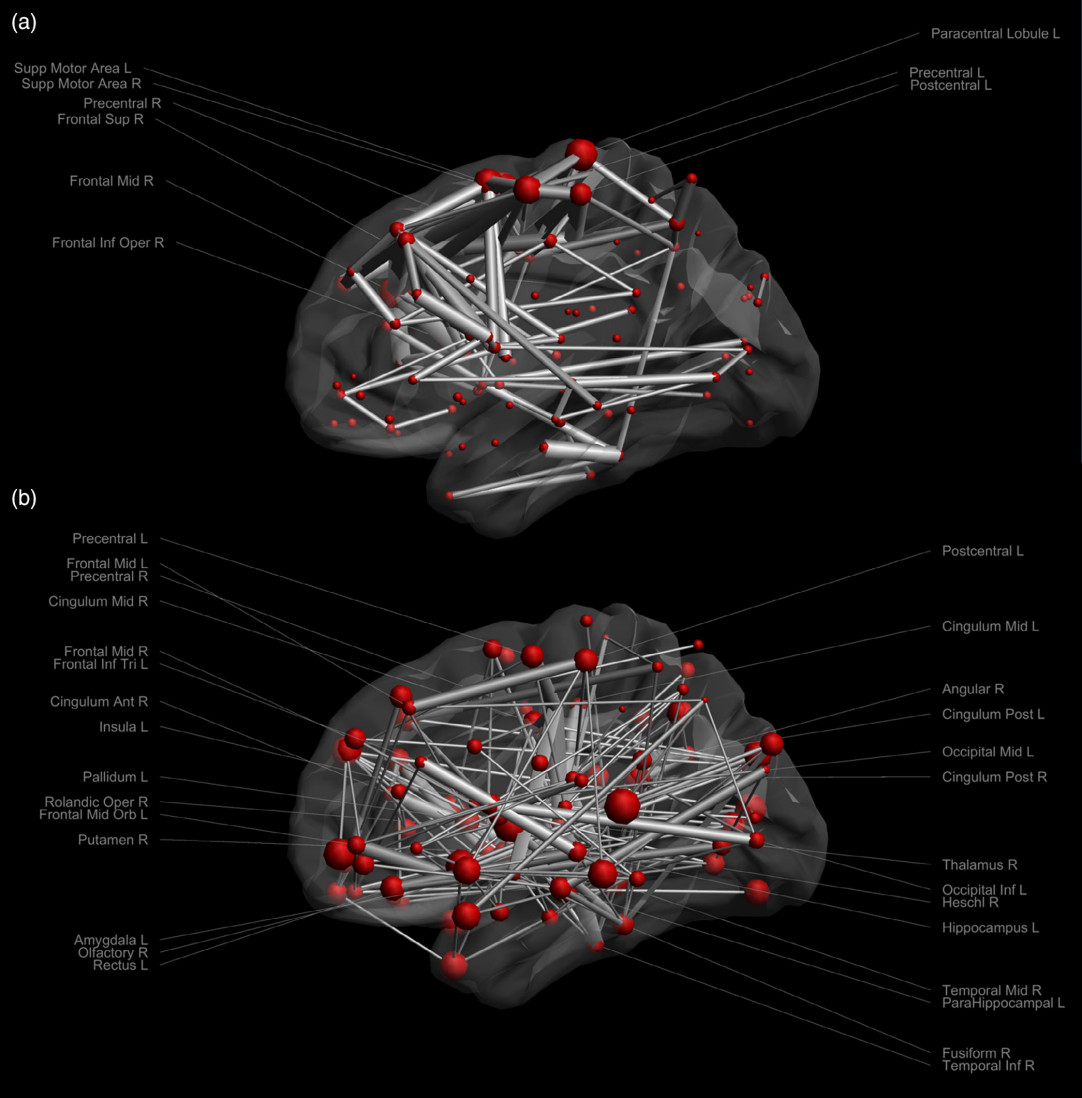
The connectivity matrix (ΔCM) of (a) streamline counts and (b) streamline length. The nodes are scaled according to the total ΔCM to every other region. The edges are scaled according to differences, thresholded as being larger than 20 in streamline counts and between 10 and 30 in streamline length. The labels of areas with an edge threshold larger than 100 in streamline counts and an edge threshold of 25–30 in streamline length are shown

Figure 8 visualizes the ΔCM in Figure 7 with the size of the nodes and edges representing the differences in streamline counts and streamline length. Node sizes reflect the sum of the differences to other brain areas. The edge size was scaled to only visualize differences larger than 20 for the streamline counts and larger than 10 (with a maximum of 30) for the streamline length for better visualization. Large differences in streamline counts can be seen in the superior area of the brain. Differences of streamline length can be found in the peripheral brain areas. These areas of large differences roughly match areas with strong gradient deviations.

## 4 DISCUSSION

In this work, we investigated the effect of gradient nonlinearities on the estimation of fiber orientations from spherical deconvolution, and evaluated their characteristics when accounting for the effective gradients in dRL. Through building a voxel‐wise deconvolution H‐matrix, dRL‐mod can consider the effective spatially varying *b*‐values and *b*‐vectors as a result of gradient field nonlinearity without interpolating the data back onto shells. By considering the voxel‐wise average *b*‐values and effective *b*‐vectors in CSD, gradient nonlinearities could be partially incorporated into the estimation. In simulations, the error of the estimated FOD peak orientations was evaluated to assess the accuracy of the spherical deconvolution methods. Overall, we found that the effect of gradient nonlinearities on fiber orientation estimates was dependent on the diffusion‐weighting, properties of the coil tensor, inherent microstructural characteristics (e.g., fiber direction and diffusivities), noise, and implementation, among others. The proposed modification of dRL FOD estimation has shown to be able to mitigate the effects of gradient field nonlinearities, while the angular accuracy following a modification of CSD estimation by using an averaged effective *b*‐value depended on the implementation and may be less affected. In in vivo experiments, angular differences between the dRL‐uni and dRL‐mod FOD estimation are shown both in HCP datasets and Connectom MRI datasets, with a comparable pattern of increasing deviations when moving away from the isocenter. Gradient nonlinearities caused different FOD characteristics and hence variations in the reconstructed fiber pathways and CMs.

### 4.1 Dependency on diffusion‐weighting, coil tensor, microstructure, and noise

The simulation study shows that when the gradient nonlinearity component along the fiber orientation is large, the bias in the FOD estimation is relatively larger (Simulations I and II). This interaction between the underlying fiber orientation and gradient deviations becomes apparent as the visibility of anatomical “features” in the synthetic brain and in vivo images of the angular deviations. For example, orientation estimates of single fiber populations in the corticospinal tract in which the primary fiber orientation is not aligned with the main gradient deviation seem to be less biased.

The effect of gradient nonlinearity on the signals also depends on diffusion weighting. The large signal changes occur at lower *b*‐values (Figure 1c), and the degree of deviation depends on the diffusivity, imposed *b*‐value and the coil tensor. The global angular deviation map of the synthetic brain derived with CSD and dRL corresponds to the pattern of gradient nonlinearity: the largest angular deviations are mainly located in the frontal lobe, the temporal lobe, and the cerebellum, which are the furthest away from the isocenter.

Noise becomes the major confounding factor in medial regions where gradient deviations are less pronounced, suggesting that the relevance of this artifact becomes more prominent in data at high SNR, which is nowadays becoming more readily available. Altogether, the effects on fiber orientation estimation are different in different datasets, that is, anatomical features can more clearly be seen in Data I than in Data II (Figure 6).

### 4.2 Dependency on spherical deconvolution implementation

CSD and dRL FOD estimation, with or without gradient nonlinearities, were compared in different fiber configurations in simulations (Figures 2, 3, 4). FOD estimation from CSD can be more robust to gradient deviations depending on the implementation. The observed differences in implementation between ExploreDTI and MRtrix (Figure S1) are further investigated in Figure S2, where we observed that differences in the regularization approach may be causing this effect. The relative robustness of CSD to gradient nonlinearities could further be due to the fitting of SH, that is, deviations in individual gradient directions may be smoothened out. The semi‐modification scheme suggested for CSD, which considers the average effective *b*‐value of all gradient directions, could ameliorate the effect of gradient nonlinearities depending on the implementation and *b*‐value (Figure S1). dRL‐mod was able to largely mitigate the effects of gradient deviations on direction‐estimates, providing similar angular deviations to dRL on signals unaffected by gradient nonlinearities. On the other hand, changes in peak magnitude were more pronounced in dRL‐mod than CSD‐mod, indicating that the effect of gradient‐nonlinearities on measures such as apparent fiber density (Raffelt et al., 2012) and hindrance modulated orientational anisotropy (Dell'Acqua, Simmons, Williams, & Catani, 2013) deserves further attention in future studies.

Previous work has shown a difference in sensitivity of the spherical deconvolution methods to the shape of the response function (Guo et al., 2019; Parker et al., 2013), where the choice of too isotropic response functions can lead to lower angular resolution in the deconvolution process, but can concomitantly mitigate spurious fibers. Gradient nonlinearities leading to lower effective *b*‐values can in some cases lead to less anisotropic response functions, especially at lower *b*‐values and crossing fibers with small separation angles. We suspect that this effect may cause the slightly lower angular accuracy of the z‐fiber in Figure 3, but with the potential benefit of avoiding spurious peaks. To further elucidate this, we show the median of angular deviations following a positive gradient deviation—leading to a sharper response function—in supplementary Table S1, Figures S1 and S5, both showing a more marked improvement with positive gradient deviations.

### 4.3 The effect of gradient nonlinearities on tractography and network analysis

The network analysis of brain connectomes relies on the accurate estimation of FODs. The FOD deviations resulting from the gradient nonlinearity in the peripheral brain areas can cause variations in network analysis (Figure 7), as measured by the differences in the CMs ΔCM. The two presented matrices, streamline count and streamline length, show high differences when comparing dRL‐uni and dRL‐mod. Differences in the number of streamlines suggest potential deviations of tractography into adjacent areas. Differences in the tract length may indicate, among others, early termination of tracking. In Figure 8, one can appreciate that brain areas showing large differences in tract counts are mainly within the frontal lobe and parietal lobe, consistent with spatial patterns of gradient deviations.

### 4.4 Implications for multishell analyses, other methods, and future work

In this work, we have focused on single‐shell analyses to be able to study the dependency on the *b*‐value and to facilitate the interpretability of the results. In contrast to the perhaps common assumption that gradient nonlinearities are the most detrimental at strong gradient strengths and high *b*‐values, our analyses suggest that fiber‐orientation estimates are also affected at lower *b*‐values where the absolute signal change per unit *b*‐value is the largest (Figure 1). Spherical deconvolution strategies have recently been extended to be compatible with multishell acquisitions, with the greatest advantage being an improved separation of tissue types and a reduction of spurious FODs in GM and CSF (Guo, Leemans, Viergever, Dell'Acqua, & de Luca, 2019; Jeurissen, Tournier, Dhollander, Connelly, & Sijbers, 2014). The effect of gradient nonlinearities on fiber orientation estimates from multishell deconvolution will depend on the *b*‐values included; if a stronger emphasis is put on lower *b*‐values our results suggest that the effect can be larger.

The simulations in this study have been designed to represent the simplest possible scenario which still allows us to systematically study effects of sufficient complexity; they assume that the response function can be described by a positive semidefinite tensor and is known a priori. Regarding the first assumption, the kernel has been parameterized in the literature by different functional forms such as zonal SH (Tournier, Calamante, Gadian, & Connelly, 2004), an axially symmetric tensor representation up to fourth order (Morez et al., 2017), and the “standard model of diffusion” (Jespersen, Kroenke, Østergaard, Ackerman, & Yablonskiy, 2007; Kroenke, Ackerman, & Yablonskiy, 2004; Novikov, Fieremans, Jespersen, & Kiselev, 2016) consisting of two axially symmetric tensors (a “Stick” with zero radial diffusivity representing intra‐axonal space and a “Zeppelin” representing extra‐axonal space). Regarding the latter model, while at sufficiently low or high *b*‐values either the extra‐ or intra‐axonal compartment dominates the signal and the response function could thus be represented by a tensor, at intermediate *b*‐values it is better represented by a weighted sum of the tensors. Future work could study this regime with other representations for the response function, but we expect that the major trends (e.g., the dependency on *b*‐value) are already captured by the simpler scenarios presented in this study.

The second assumption of an a priori known response function is common to several spherical deconvolution strategies. dRL has shown to be less sensitive to the miscalibration of the response function than CSD (Parker et al., 2013). The response function is commonly described by one of the aforementioned representations (e.g., tensor, SH), and the parameters of this representation can either be chosen by the user or calibrated from the data. To account for spatially varying b‐matrices due to gradient nonlinearities, the response function representation should both have an angular and radial component. Current state‐of‐the‐art implementations for data‐driven calibration of a global WM response function (Tax, Jeurissen, Viergever, & Leemans, 2013; Tournier et al., 2012) rely on the zonal SH representation of the response function, and need to be adapted to other representations to be compatible with data that is not sampled on shells (Morez et al., 2017).

In addition, various methods have been proposed to estimate the kernel voxel‐wise (de Almeida Martins et al., 2019; Kaden et al., 2007; Novikov et al., 2018; Schultz & Groeschel, 2013). The majority of these approaches first factors out the orientational dependency by taking the “powder average” or “spherical mean” to estimate the kernel. This is however problematic in the case of gradient nonlinearities, as the effective sampling is not on a sphere and the spherical mean will thus be biased. Estimation strategies that attempt to estimate the kernel and the FOD simultaneously without first factoring out the orientational dependency are better suited to deal with gradient nonlinearities (Jespersen et al., 2010; Neto Henriques, Tax, Shemesh, & Veraart, 2019). The effects on fiber orientation estimates found in this study, where the response function is assumed to be known, could thus be translated to such approaches, in which the estimated kernel approaches the true one.

## 5 CONCLUSION

In this study, we explored the sensitivity of spherical deconvolution approaches, dRL and CSD, to gradient nonlinearity effects in diffusion MRI. CSD can be more robust to gradient nonlinearities, depending on the implementation. In the proposed dRL‐mod framework, which does not require interpolation of the data, we explored whether employing the effective *b*‐value and *b*‐vector improved the estimation of fiber orientation. Numerical simulations, a synthetic brain, HCP datasets, and Connectom MRI datasets were used in this work. By comparing the FOD peak orientations with and without applying the effective gradients, we found that knowledge on the gradient nonlinearities can be used within the dRL scheme to reduce angular errors. The angular deviations of the synthetic brain and in vivo data show similar patterns as the Frobenius norm of the gradient coil tensor field, and gradient nonlinearities can affect both low *b*‐value and high *b*‐value acquisitions. In datasets with relatively high SNR, anatomical structures appear in the angular deviation maps, indicating that the deviations are also depending on the angles between the gradient direction and the underlying fiber orientation. Large negative gradient deviations can affect the sharpness of the response function when accounted for, slightly reducing angular accuracy in a balanced trade‐off with reducing spurious peaks in agreement with previous studies. Finally, changes in CMs when not considering the gradient nonlinearity effects highlight potential detrimental effects in tractography and network‐based applications of diffusion MRI.

## Supporting information


**FIGURE S1** The distribution of the angular deviations of FOD peaks from ExploreDTI and MRtrix CSD estimation, with a fixed gradient deviation of Δ***L*** = diag([−0.13 –0.14 –0.05]) and Δ***L*** = diag([0.13 0.14 0.05]), at b = 3,000 s/mm^2^ and b = 1,000 s/mm^2^. The simulated fiber orientation is along the x‐axis. The default settings in the regularization process were used in ExploreDTI and MRtrix.

**FIGURE S2** CSD FOD peak angular deviations with different regularization parameters in the ExploreDTI and MRtrix, in presence of gradient nonlinearities Δ***L*** = diag([−0.13–0.14 –0.05]). EDTI: ExploreDTI. Lambda and tau are parameters used in the regularization step. MRtrix negL: neg_lambda; MRtrix normL: norm_lambda. Details of the MRtrix parameter settings can be found at the software developer's websites (https://mrtrix.readthedocs.io/en/latest/reference/commands/dwi2fod.html)

**FIGURE S3** The angular deviations of FOD peaks with gradient deviations of Δ***L*** = diag([−0.2–0.1 0]) in x‐, y‐ and z‐ axes, at SNR 30 of a fixed fiber orientation along y‐ direction (simulation III), b = 3,000 s/mm^2^, in addition to the results of Δ*L* shown in Figure 3. dRL‐uni no dev: dRL‐uni estimation without the gradient deviation Δ***L***; dRL‐uni dev: dRL‐uni estimation with the gradient deviation Δ***L***; dRL‐mod dev: dRL‐mod estimation with the gradient deviation Δ***L***; CSD‐uni no dev: CSD‐uni estimation without the gradient deviation Δ***L***; CSD‐uni dev: CSD‐uni estimation with the gradient deviation Δ***L***; CSD‐mod dev: CSD‐mod estimation with the gradient deviation Δ***L***.

**FIGURE S4** The angular deviations of FOD peaks with gradient deviations of Δ***L*** = diag([−0.13–0.14 –0.05]) in x‐, y‐ and z‐ axes of a fixed fiber orientation along y‐ axis at different SNRs, b = 3,000 s/mm^2^. More deviations of FOD peak orientations can be spotted when the gradient deviations are present in the dRL‐uni estimation, for all the SNR levels in simulation IV. dRL‐uni no dev: dRL‐uni estimation without the gradient deviation Δ***L***; dRL‐uni dev: dRL‐uni estimation with the gradient deviation Δ***L***; dRL‐mod dev: dRL‐mod estimation with the gradient deviation Δ***L***; CSD‐uni no dev: CSD‐uni estimation without the gradient deviation Δ***L***; CSD‐uni dev: CSD‐uni estimation with the gradient deviation Δ***L***; CSD‐mod dev: CSD‐mod estimation with the gradient deviation Δ***L***.

**FIGURE S5** The angular deviations of crossing fibers with gradient deviations of with a different sign of the diagonal elements in Δ*L* in comparison to Figure 4, Δ***L*** = diag([0.15 0.15 0.15]) (simulation V), b = 3,000 s/mm^2^. θ stands for the simulated separation angles of the crossing fibers. dRL‐uni no dev: dRL‐uni estimation without the gradient deviation Δ***L***; dRL‐uni dev: dRL‐uni estimation with the gradient deviation Δ***L***; dRL‐mod dev: dRL‐mod estimation with the gradient deviation Δ***L***; CSD‐uni no dev: CSD‐uni estimation without the gradient deviation Δ***L***; CSD‐uni dev: CSD‐uni estimation with the gradient deviation Δ***L***; CSD‐mod dev: CSD‐mod estimation with the gradient deviation Δ***L***.

**FIGURE S6** FOD peak amplitudes of single fibers with gradient deviations of Δ***L*** = diag([−0.15–0.15 –0.15]) and Δ***L*** = diag([0.15 0.15 0.15]), b = 3,000 s/mm^2^. dRL‐uni no dev: dRL‐uni estimation without the gradient deviation Δ***L***; dRL‐uni dev: dRL‐uni estimation with the gradient deviation Δ***L***; dRL‐mod dev: dRL‐mod estimation with the gradient deviation Δ***L***; CSD‐uni no dev: CSD‐uni estimation without the gradient deviation Δ***L***; CSD‐uni dev: CSD‐uni estimation with the gradient deviation Δ***L***; CSD‐mod dev: CSD‐mod estimation with the gradient deviation Δ***L***.

**FIGURE S7** FOD peak amplitudes of crossing fibers with gradient deviations of Δ***L*** = diag([−0.15–0.15 –0.15]) and Δ***L*** = diag([0.15 0.15 0.15]), b = 3,000 s/mm^2^. θ stands for the simulated separation angles of the crossing fibers. Dashed lines indicate the simulated signal fractions of the fiber populations. dRL‐uni no dev: dRL‐uni estimation without the gradient deviation Δ***L***; dRL‐uni dev: dRL‐uni estimation with the gradient deviation Δ***L***; dRL‐mod dev: dRL‐mod estimation with the gradient deviation Δ***L***; CSD‐uni no dev: CSD‐uni estimation without the gradient deviation Δ***L***; CSD‐uni dev: CSD‐uni estimation with the gradient deviation Δ***L***; CSD‐mod dev: CSD‐mod estimation with the gradient deviation Δ***L***.

**TABLE S1** The median of the angular deviations with various settings (Simulation I and Simulation II). Δ***L*** +: Δ***L*** **=** diag([0.13 0.14 0.05]); Δ***L*** ‐: Δ***L*** = diag([−0.13 –0.14 –0.05]).

## Data Availability

The authors confirm that the data supporting the findings of this study is available from public databases. The in vivo datasets of HCP are available at: https://www.humanconnectome.org/study/hcp-young-adult/data-releases. The in vivo datasets of CDMRI challenge are available at: https://projects.iq.harvard.edu/cdmri2018/data.
