# Supplementary figures and images for: The effect of gradient nonlinearities on fiber orientation estimates from spherical deconvolution of diffusion magnetic resonance imaging data
Source: Hum Brain Mapp. 2020 Oct 9;42(2):367–83. doi: 10.1002/hbm.25228 (PMC7776002; doi:10.1002/hbm.25228)

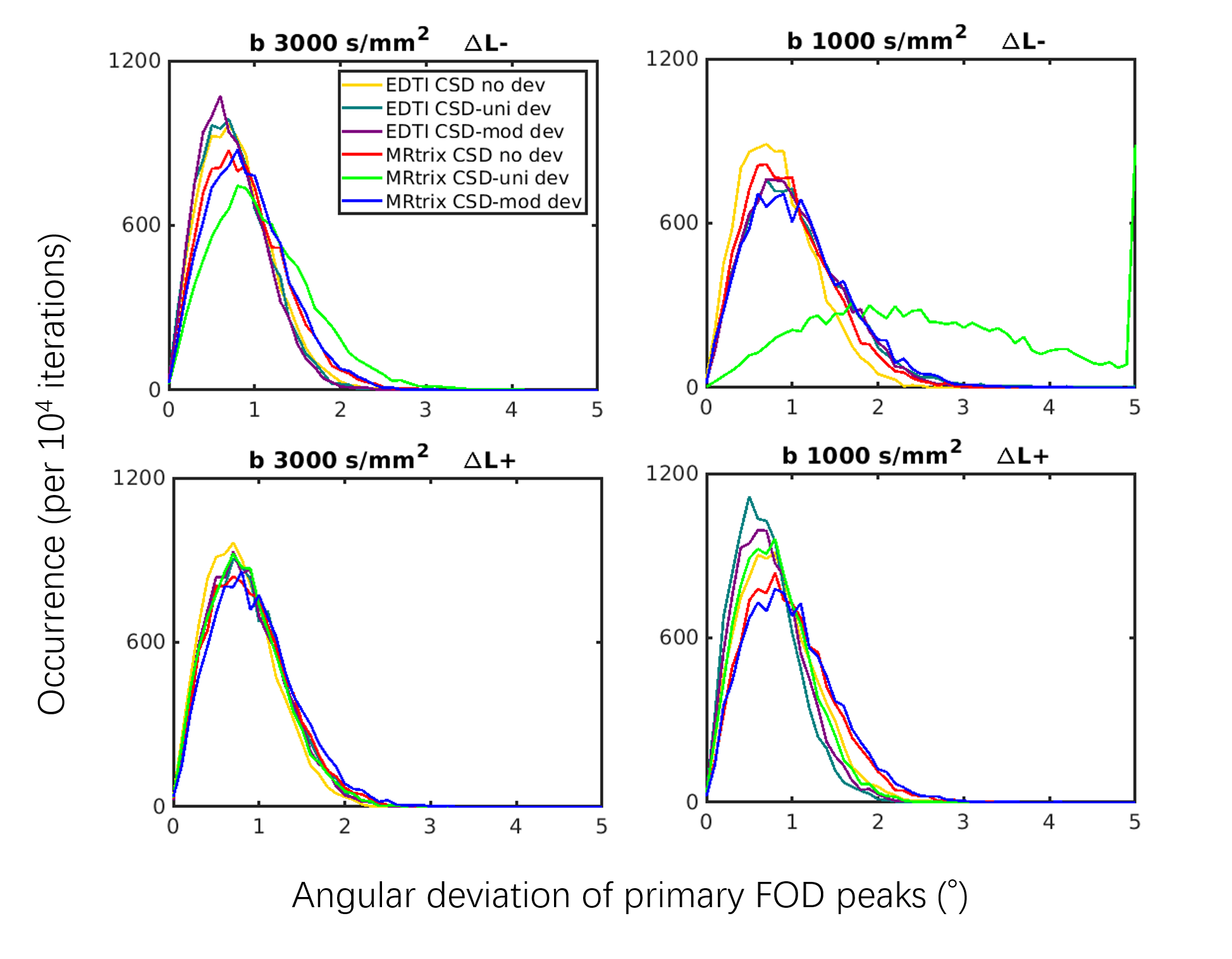

Supplement: Supplementary file 1 — FIGURE S1 The distribution of the angular deviations of FOD peaks from ExploreDTI and MRtrix CSD estimation, with a fixed gradient deviation of ΔL = diag([−0.13 –0.14 –0.05]) and ΔL = diag([0.13 0.14 0.05]), at b = 3,000 s/mm2 and b = 1,000 s/mm2. The simulated fiber orientation is along the x‐axis. The default settings in the regularization process were used in ExploreDTI and MRtrix. [file HBM-42-367-s001.tif]

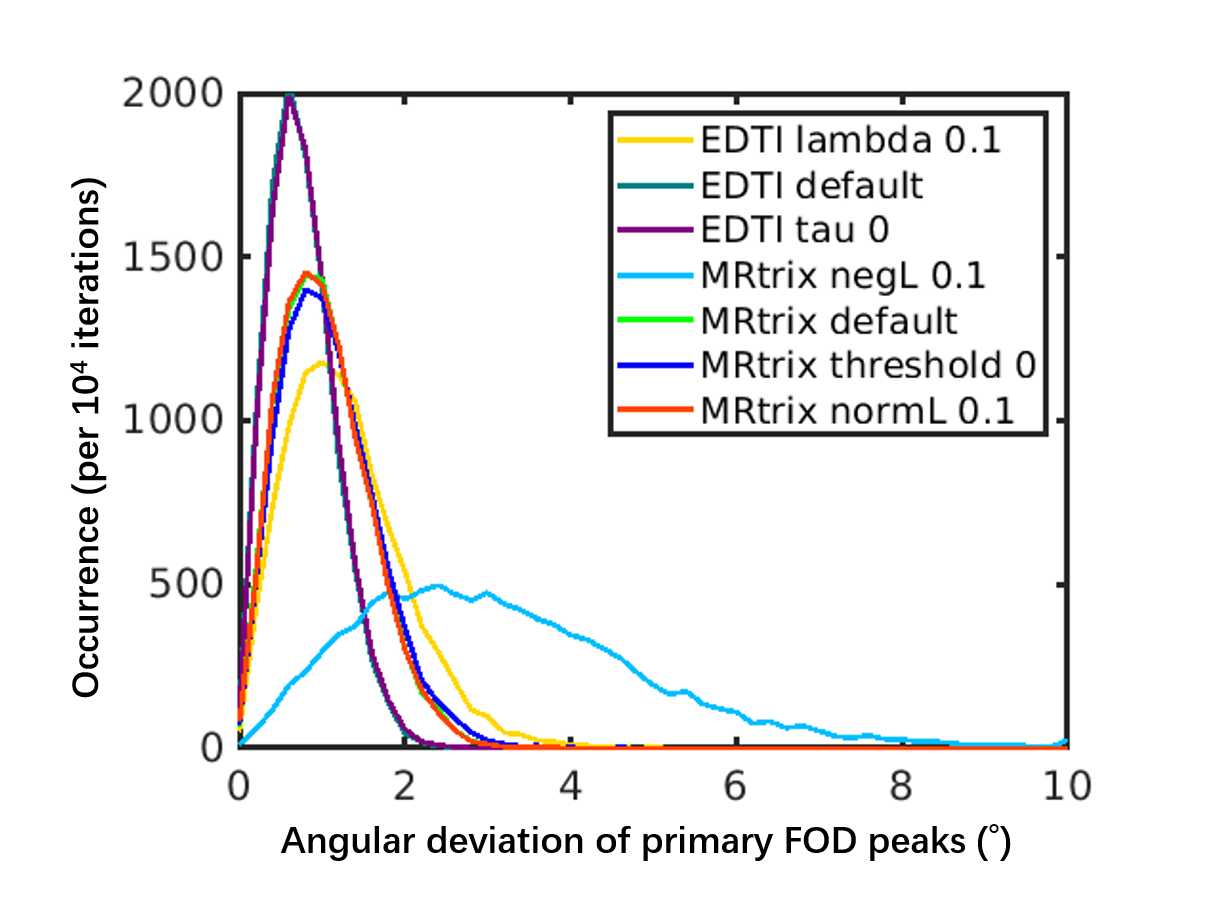

Supplement: Supplementary file 2 — FIGURE S2 CSD FOD peak angular deviations with different regularization parameters in the ExploreDTI and MRtrix, in presence of gradient nonlinearities ΔL = diag([−0.13–0.14 –0.05]). EDTI: ExploreDTI. Lambda and tau are parameters used in the regularization step. MRtrix negL: neg_lambda; MRtrix normL: norm_lambda. Details of the MRtrix parameter settings can be found at the software developer's websites (https://mrtrix.readthedocs.io/en/latest/reference/commands/dwi2fod.html) [file HBM-42-367-s002.tif]

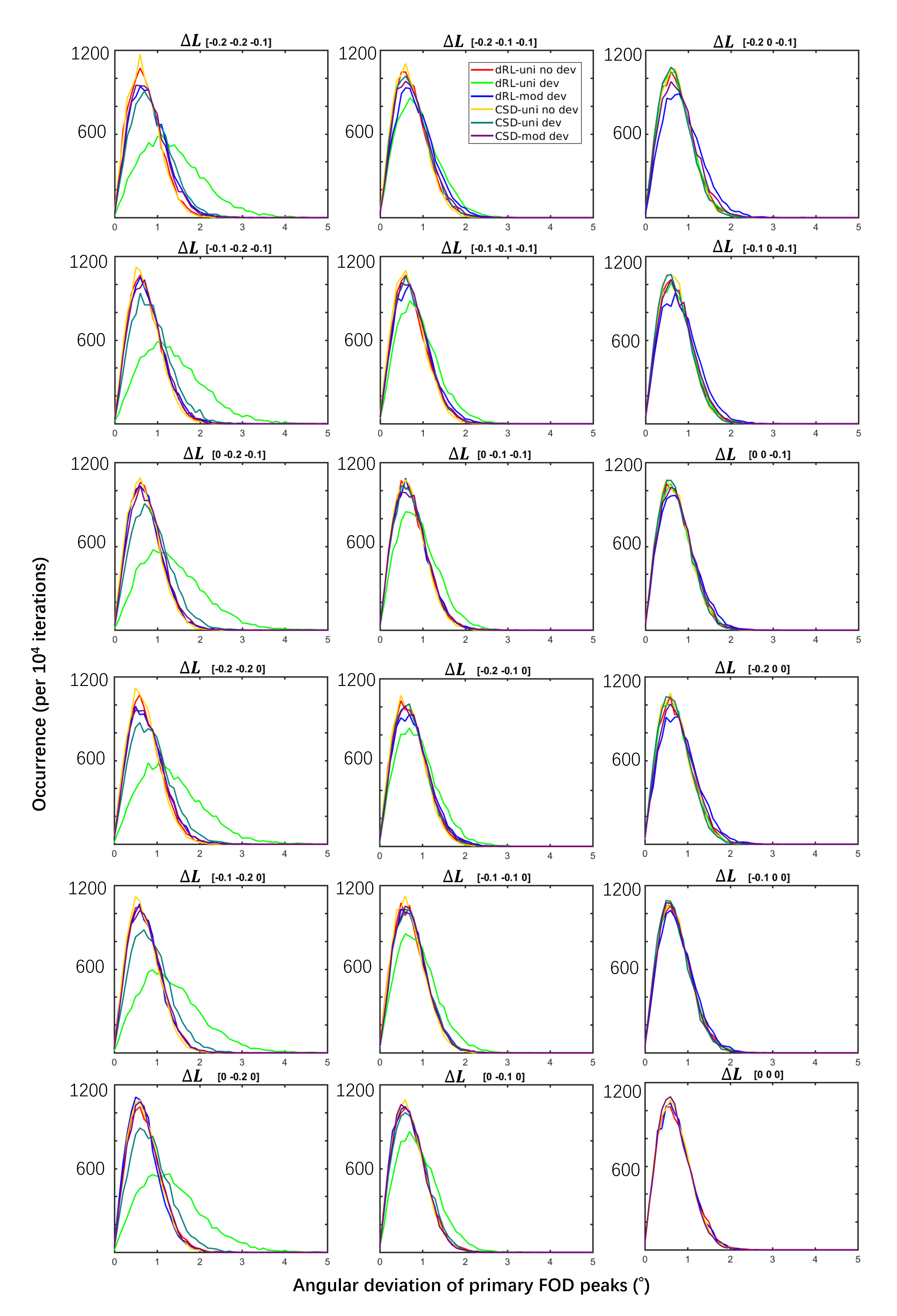

Supplement: Supplementary file 3 — FIGURE S3 The angular deviations of FOD peaks with gradient deviations of ΔL = diag([−0.2–0.1 0]) in x‐, y‐ and z‐ axes, at SNR 30 of a fixed fiber orientation along y‐ direction (simulation III), b = 3,000 s/mm2, in addition to the results of ΔL shown in Figure 3. dRL‐uni no dev: dRL‐uni estimation without the gradient deviation ΔL; dRL‐uni dev: dRL‐uni estimation with the gradient deviation ΔL; dRL‐mod dev: dRL‐mod estimation with the gradient deviation ΔL; CSD‐uni no dev: CSD‐uni estimation without the gradient deviation ΔL; CSD‐uni dev: CSD‐uni estimation with the gradient deviation ΔL; CSD‐mod dev: CSD‐mod estimation with the gradient deviation ΔL. [file HBM-42-367-s003.tif]

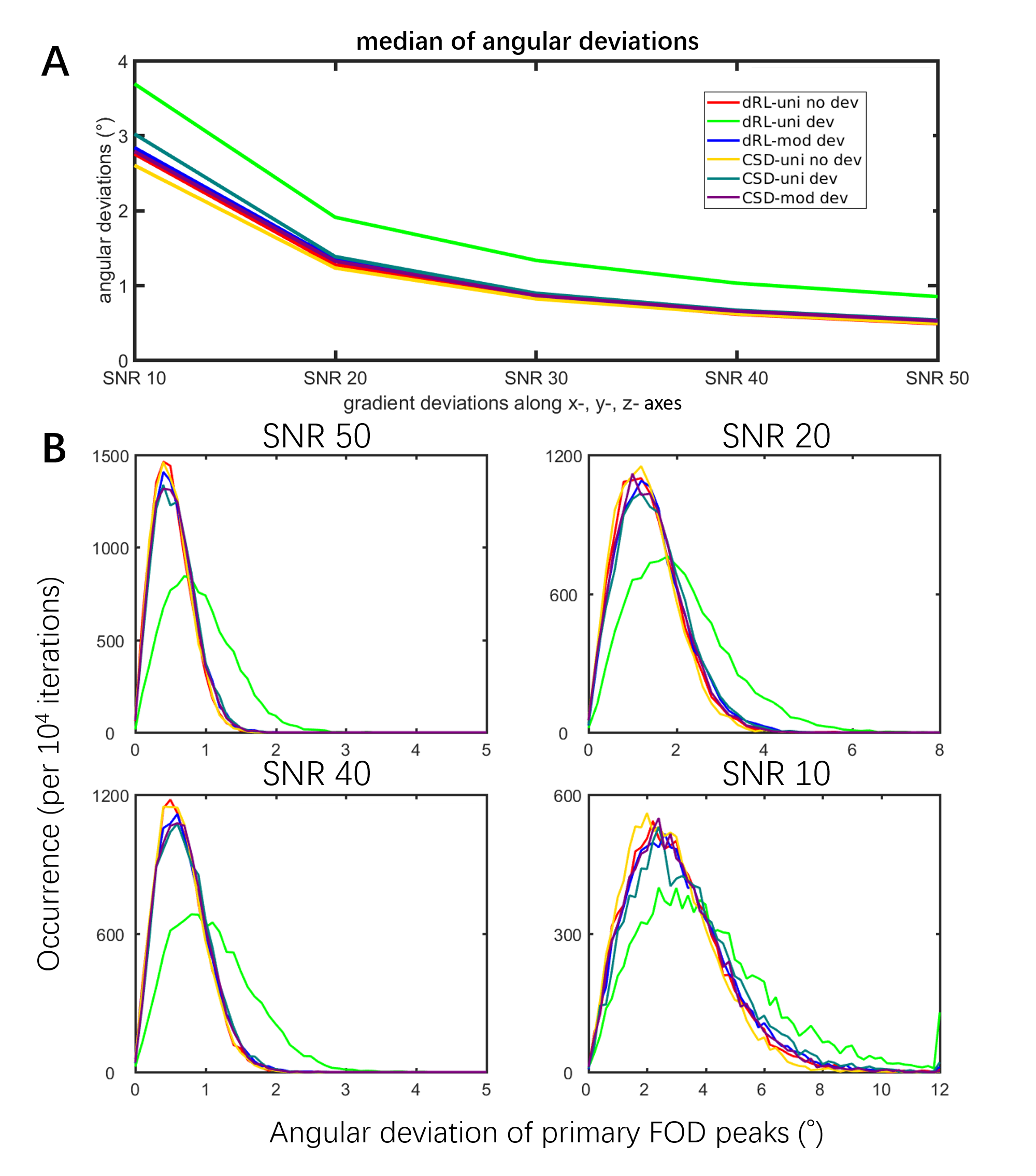

Supplement: Supplementary file 4 — FIGURE S4 The angular deviations of FOD peaks with gradient deviations of ΔL = diag([−0.13–0.14 –0.05]) in x‐, y‐ and z‐ axes of a fixed fiber orientation along y‐ axis at different SNRs, b = 3,000 s/mm2. More deviations of FOD peak orientations can be spotted when the gradient deviations are present in the dRL‐uni estimation, for all the SNR levels in simulation IV. dRL‐uni no dev: dRL‐uni estimation without the gradient deviation ΔL; dRL‐uni dev: dRL‐uni estimation with the gradient deviation ΔL; dRL‐mod dev: dRL‐mod estimation with the gradient deviation ΔL; CSD‐uni no dev: CSD‐uni estimation without the gradient deviation ΔL; CSD‐uni dev: CSD‐uni estimation with the gradient deviation ΔL; CSD‐mod dev: CSD‐mod estimation with the gradient deviation ΔL. [file HBM-42-367-s004.tif]

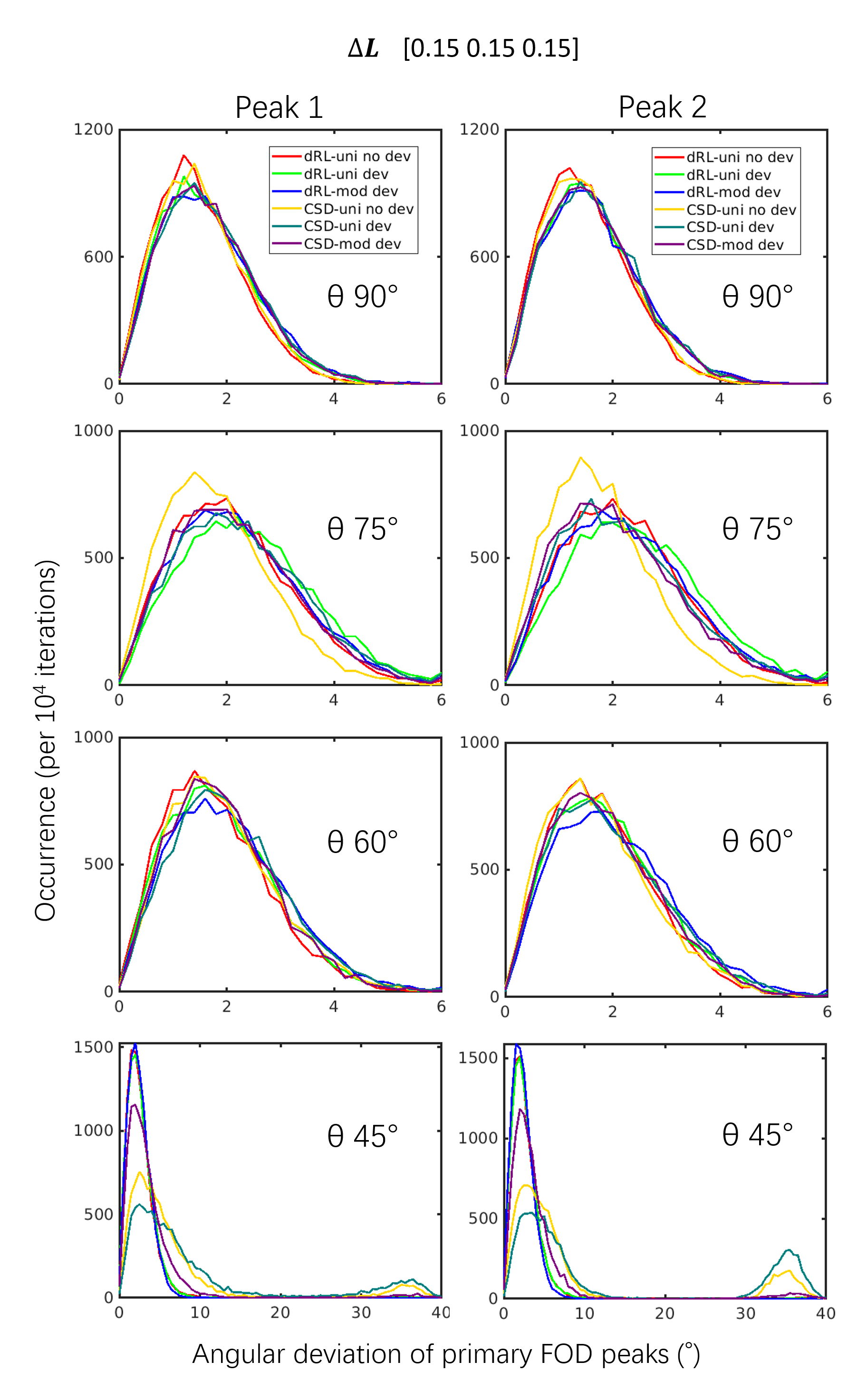

Supplement: Supplementary file 5 — FIGURE S5 The angular deviations of crossing fibers with gradient deviations of with a different sign of the diagonal elements in ΔL in comparison to Figure 4, ΔL = diag([0.15 0.15 0.15]) (simulation V), b = 3,000 s/mm2. θ stands for the simulated separation angles of the crossing fibers. dRL‐uni no dev: dRL‐uni estimation without the gradient deviation ΔL; dRL‐uni dev: dRL‐uni estimation with the gradient deviation ΔL; dRL‐mod dev: dRL‐mod estimation with the gradient deviation ΔL; CSD‐uni no dev: CSD‐uni estimation without the gradient deviation ΔL; CSD‐uni dev: CSD‐uni estimation with the gradient deviation ΔL; CSD‐mod dev: CSD‐mod estimation with the gradient deviation ΔL. [file HBM-42-367-s005.tif]

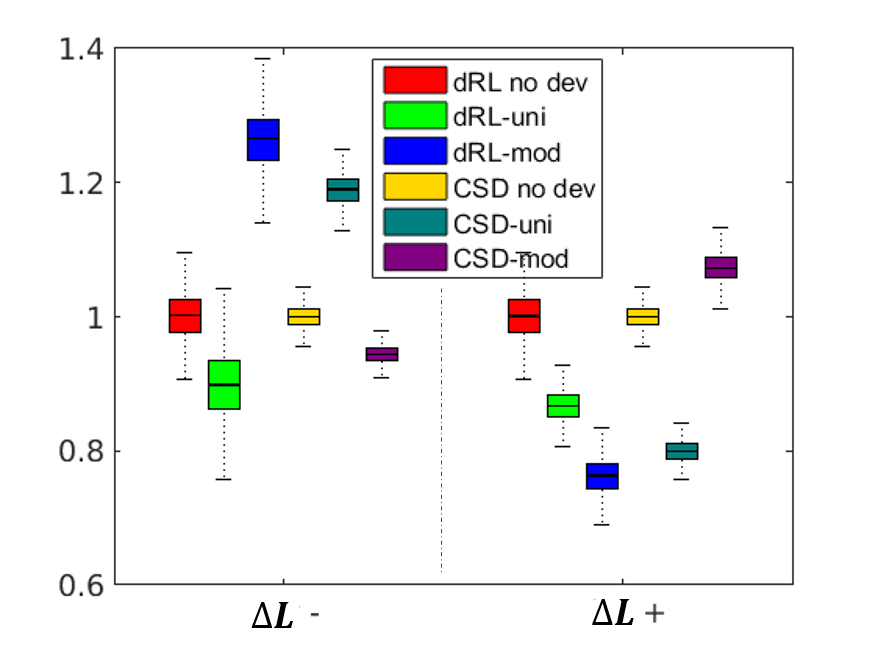

Supplement: Supplementary file 6 — FIGURE S6 FOD peak amplitudes of single fibers with gradient deviations of ΔL = diag([−0.15–0.15 –0.15]) and ΔL = diag([0.15 0.15 0.15]), b = 3,000 s/mm2. dRL‐uni no dev: dRL‐uni estimation without the gradient deviation ΔL; dRL‐uni dev: dRL‐uni estimation with the gradient deviation ΔL; dRL‐mod dev: dRL‐mod estimation with the gradient deviation ΔL; CSD‐uni no dev: CSD‐uni estimation without the gradient deviation ΔL; CSD‐uni dev: CSD‐uni estimation with the gradient deviation ΔL; CSD‐mod dev: CSD‐mod estimation with the gradient deviation ΔL. [file HBM-42-367-s006.tif]

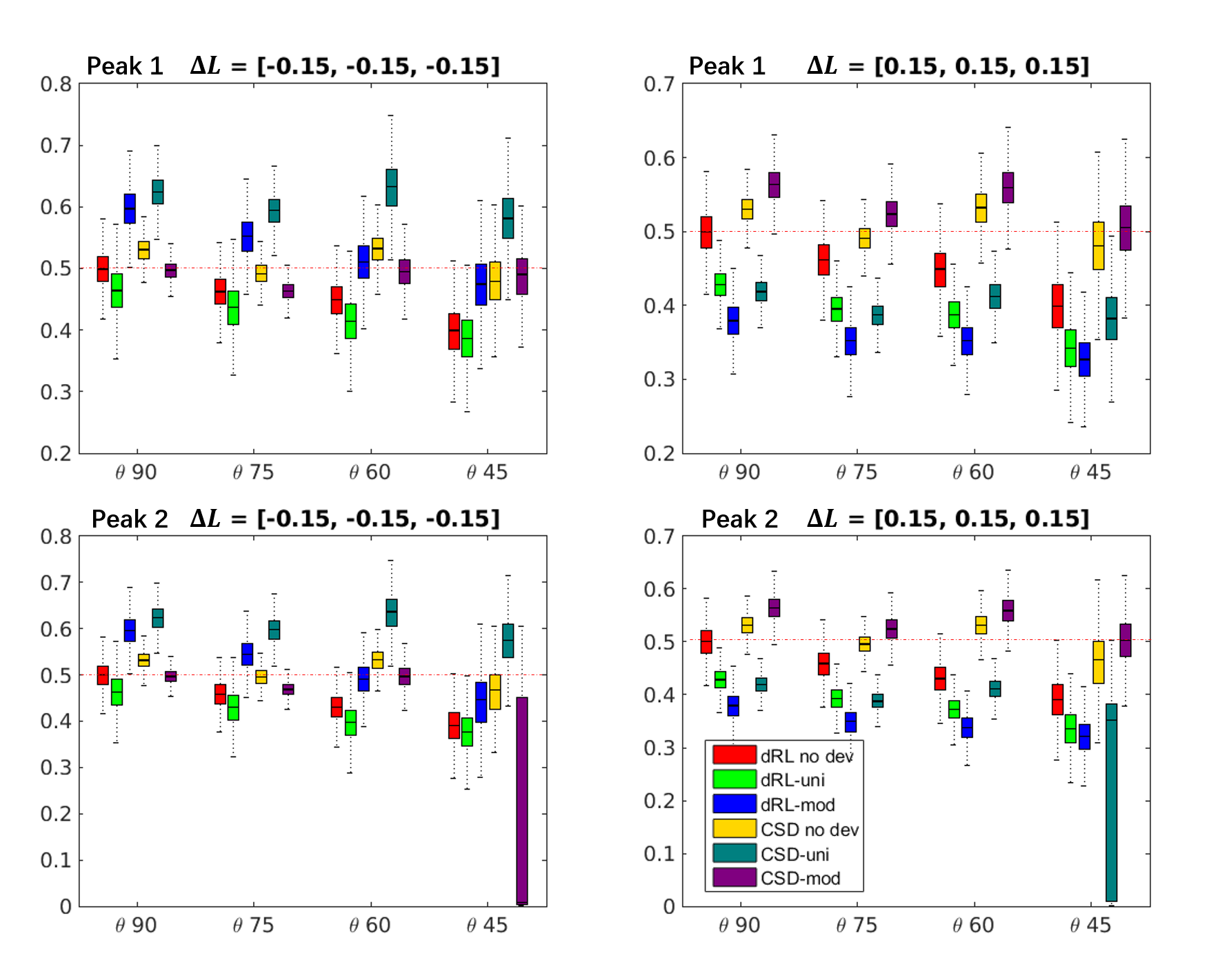

Supplement: Supplementary file 7 — FIGURE S7 FOD peak amplitudes of crossing fibers with gradient deviations of ΔL = diag([−0.15–0.15 –0.15]) and ΔL = diag([0.15 0.15 0.15]), b = 3,000 s/mm2. θ stands for the simulated separation angles of the crossing fibers. Dashed lines indicate the simulated signal fractions of the fiber populations. dRL‐uni no dev: dRL‐uni estimation without the gradient deviation ΔL; dRL‐uni dev: dRL‐uni estimation with the gradient deviation ΔL; dRL‐mod dev: dRL‐mod estimation with the gradient deviation ΔL; CSD‐uni no dev: CSD‐uni estimation without the gradient deviation ΔL; CSD‐uni dev: CSD‐uni estimation with the gradient deviation ΔL; CSD‐mod dev: CSD‐mod estimation with the gradient deviation ΔL. [file HBM-42-367-s007.tif]

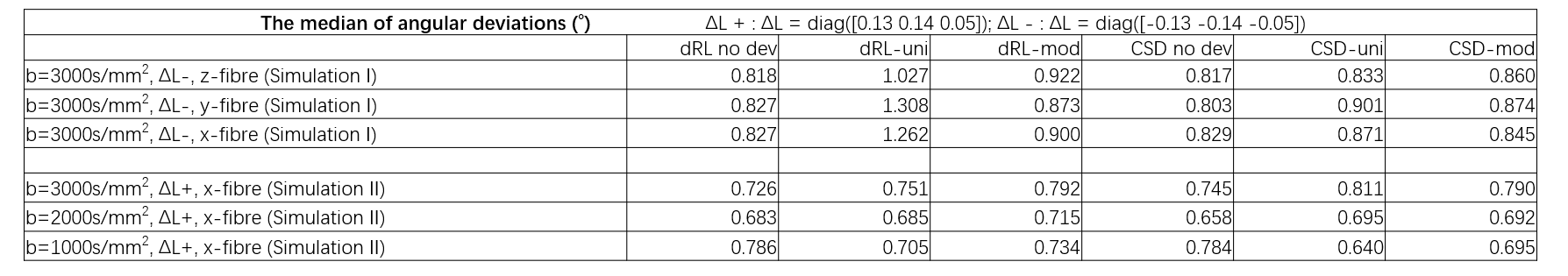

Supplement: Supplementary file 8 — TABLE S1 The median of the angular deviations with various settings (Simulation I and Simulation II). ΔL +: ΔL = diag([0.13 0.14 0.05]); ΔL ‐: ΔL = diag([−0.13 –0.14 –0.05]). [file HBM-42-367-s008.tif]
